# Insights into Enzymatic Catalysis from Binding and Hydrolysis of Diadenosine Tetraphosphate by *E*. *coli* Adenylate Kinase

**DOI:** 10.1021/acs.biochem.3c00189

**Published:** 2023-07-07

**Authors:** Sonja Tischlik, Melanie Oelker, Per Rogne, A. Elisabeth Sauer-Eriksson, Malte Drescher, Magnus Wolf-Watz

**Affiliations:** † Department of Chemistry, Konstanz Research School Chemical Biology, University of Konstanz, 78457 Konstanz, Germany; ‡ Department of Chemistry, Umeå University, SE-901 87 Umeå, Sweden; § Centre of Microbial Research (UCMR), Umeå University, SE-901 87 Umeå, Sweden

## Abstract

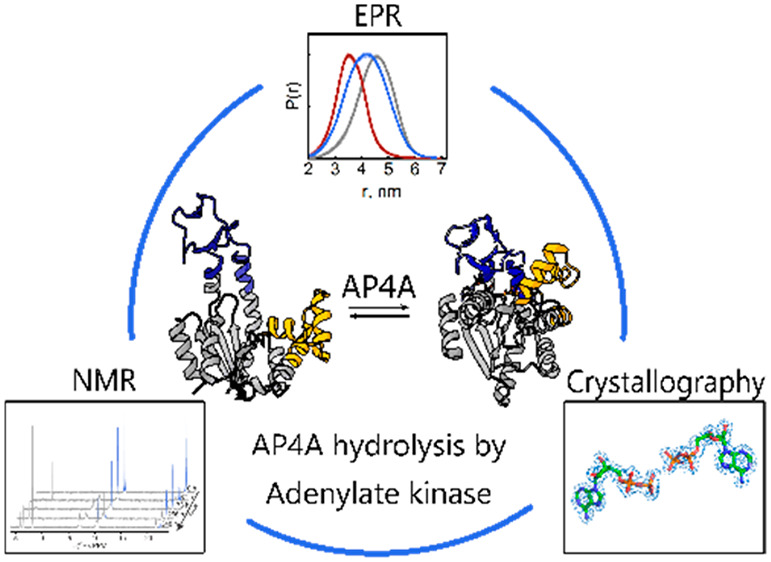

Adenylate kinases play a crucial role in cellular energy homeostasis through the interconversion of ATP, AMP, and ADP in all living organisms. Here, we explore how adenylate kinase (AdK) from *Escherichia coli* interacts with diadenosine tetraphosphate (AP4A), a putative alarmone associated with transcriptional regulation, stress, and DNA damage response. From a combination of EPR and NMR spectroscopy together with X-ray crystallography, we found that AdK interacts with AP4A with two distinct modes that occur on disparate time scales. First, AdK dynamically interconverts between open and closed states with equal weights in the presence of AP4A. On a much slower time scale, AdK hydrolyses AP4A, and we suggest that the dynamically accessed substrate-bound open AdK conformation enables this hydrolytic activity. The partitioning of the enzyme into open and closed states is discussed in relation to a recently proposed linkage between active site dynamics and collective conformational dynamics.

Despite being the most extensively studied dinucleotide phosphate, the role of diadenosine *P*^1^,*P*^4^-di(adenosine-5′-)tetraphosphate (AP4A) is yet unclear.^1,2^ Cells of all kingdoms of life have mechanisms for the synthesis and degradation of AP4A. Synthesis can be performed by aminoacetyl-tRNA-synthetases, DNA and RNA ligases, acyl-coenzyme A synthetases, ubiquitin-activating enzymes, and others.^1,3^ Phosphorylases and hydrolases degrade AP4A by splitting the molecule asymmetrically or symmetrically.^1,3,4^

A function as an extracellular messenger was proposed for AP4A in the nervous, ocular, and cardiovascular system.^5^ In bacteria, AP4A was suggested as a precursor for RNA capping^6^—indicating a role in transcriptional regulation—and studied in the context of DNA damage response.^1^

During stress, the concentration of AP4A increases between 2 to 100-fold, depending on cell type, from its normal nano- to micromolar concentration.^1^ Consequently, AP4A was suggested to be either a stress metabolite or an alarmone, a secondary messenger for stress.^1,2^

AP4A can bind ATP-binding proteins where it competes for the ATP binding pockets because of the similar chemical structure.^1,2^ An even closer analogue is the adenylate kinase (AdK) inhibitor *P*^1^,*P*^5^-di(adenosine-5′-)pentaphosphate (AP5A),^7^ with one extra phosphate group compared with AP4A (Figure 1). This suggests that AP4A may interact with AdK, and AP4A has, indeed, been found to bind *E*. *coli* AdK with micromolar affinity.^8^

**Figure 1:**
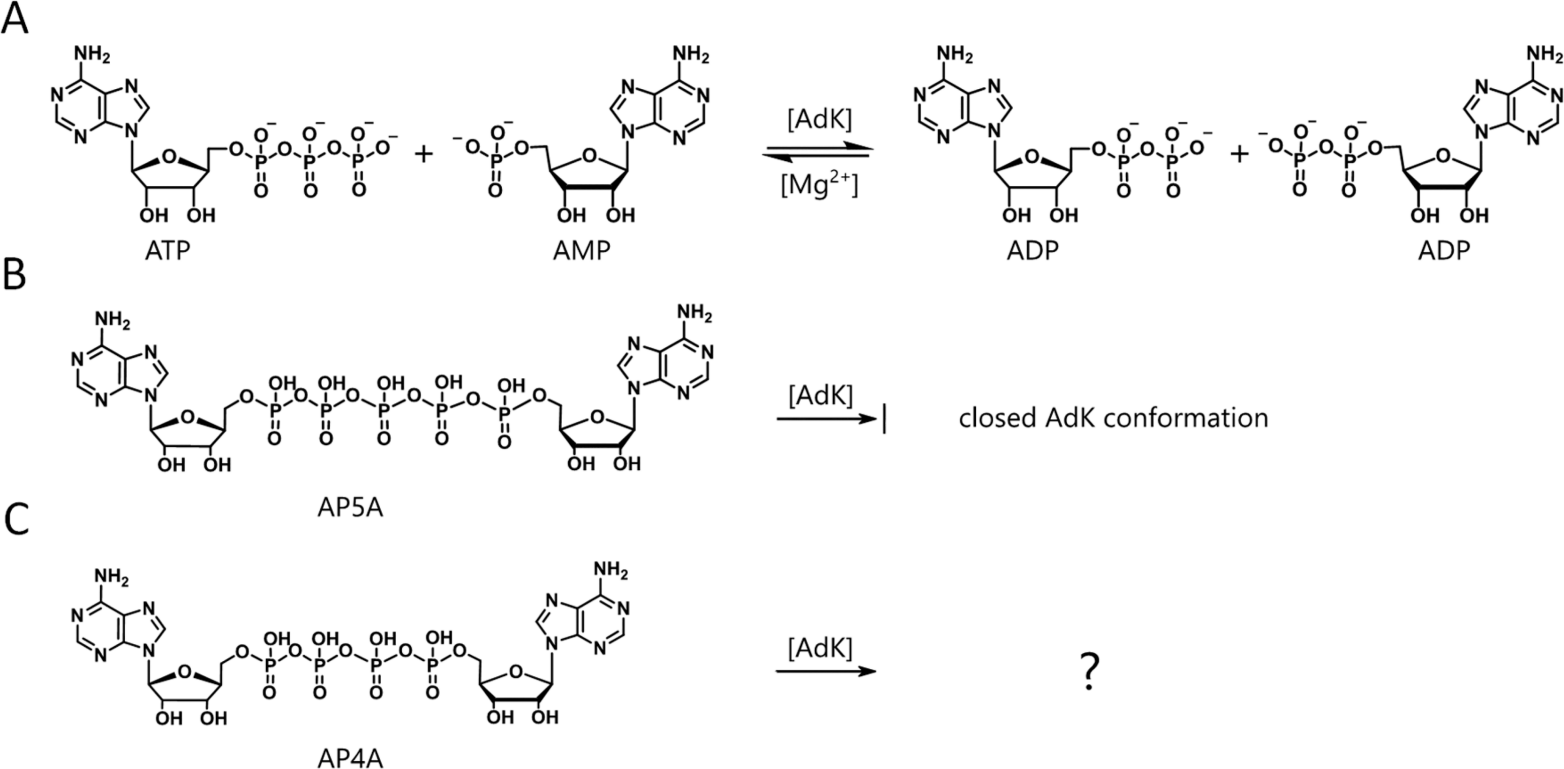
Chemical structures of AdK substrates and inhibitors. (A) AdK interconversion of its substrates ATP and AMP into two ADP with Mg^2+^ as cofactor. (B) The inhibitor AP5A that leads to a closed enzyme conformation. (C) AP4A (studied here).

AdK is a housekeeping enzyme that is present in all kingdoms of life.^9^ It maintains the cellular energy homeostasis by catalyzing the reversible Mg^2+^-dependent interconversion of ATP and AMP to two ADPs.^10,11^ AdK is well studied and serves as one of the principal model systems for enzyme dynamics.^10,12−17^

*Escherichia coli (E*. *coli)* AdK has three subdomains: a core and two flexible substrate-binding subdomains, denoted ATPlid and AMPbd. The latter two domains fold onto the core region upon substrate binding, thereby leading to an overall more compact conformation.^11^ A compact, closed conformation can also be induced when the inhibitor AP5A binds.^7^ So far, AP4A was thought to be an AdK inhibitor, like AP5A.^8^

In order to obtain molecular insights into the interaction between AP4A and *E*. *coli* AdK, we have undertaken a study on the basis of X-ray crystallography, electron paramagnetic resonance (EPR), and nuclear magnetic resonance (NMR) spectroscopy. We found that in contrast to AP5A binding, AdK remains active when bound to AP4A and is able to hydrolyze AP4A.

First, we confirmed AP4A binding to *E*. coli AdK,^8^ and the dissociation constant (*K*_d_) was found to be 14 μM (Figure S1). This dissociation constant is lower (i.e., stronger binding) than that of ATP (*K*_d_ ≈ 50 μM),^18^ but higher than that of AP5A (*K*_d_ ≈ 140–350 nM).^19,20^ The increased binding affinity relative to ATP suggests that both adenosine bases of AP4A interact with the enzyme, while the decreased affinity compared with AP5A indicates that shortening the AP5A molecule by one phosphate group might introduce strain that contributes to the reduced affinity.

To assess the structure of AP4A-bound AdK, we cocrystallized the enzyme with AP4A and determined the X-ray structure to a final resolution of 1.49 Å (Figure 2, Table S2, PDB ID: 8CRG). Surprisingly, the crystals trapped AdK in the closed conformation in complex with two ADP molecules (Figure S3A). Attempts to model AP4A as a ligand could not sufficiently explain the obtained electron density, as it leads to unusual bond angles and lengths in AP4A (Supporting Information section 3.2 and Figures S2 and S3). Instead, modeling of two ADP molecules explains the observed electron density considerably better (Figure 2). Thus, we conclude that two ADPs rather than one AP4A molecule is bound to AdK in the crystal.

**Figure 2:**
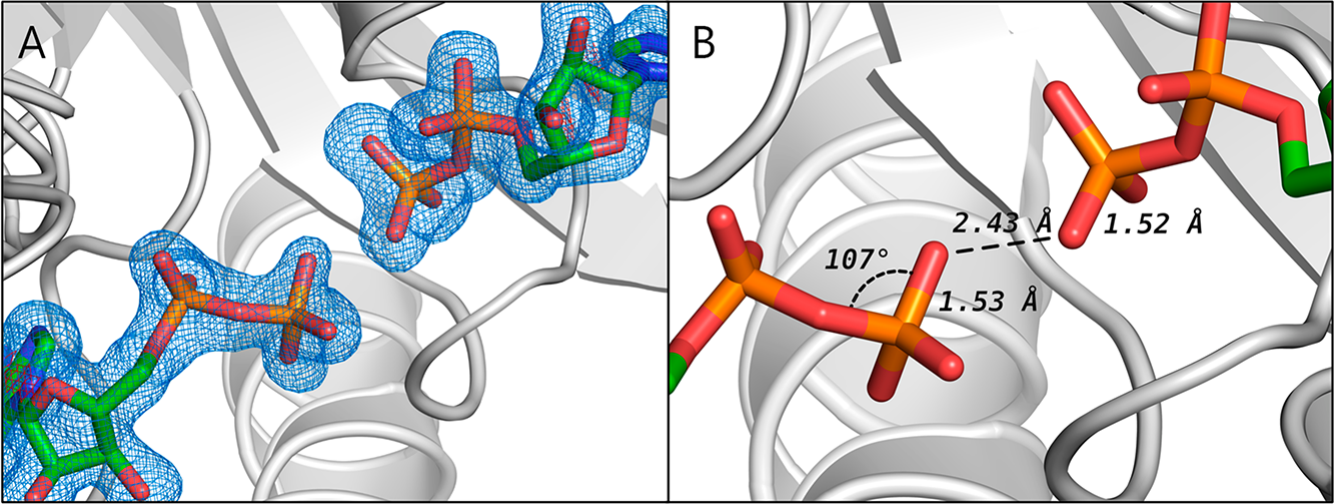
X-ray structure of *E*. *coli* AdK cocrystallized with AP4A. (A) Two ADP molecules modeled into the ligand electron density. The Fo-Fc omit electron density map (blue) is contoured at 3 σ. (B) Magnification of the β-phosphate group of ADP with an exemplary O–P–O angle and distances.

Considering that neither ADP nor other mononucleotides were added for the crystallization, the source of ADP is attributed to AdK-mediated hydrolysis of AP4A. The rate of AP4A hydrolysis by wild-type AdK was determined with ^31^P NMR to *k*_cat_ ≈ 5 × 10^–6^ s^–1^ with Mg^2+^ present at 295 K (Figure 3, pink). We excluded nonenzymatic hydrolysis, since AP4A was stable over at least 2 weeks under the experimental conditions in the absence of the enzyme (Figure 3B,C, black). The rate of AP4A hydrolysis is much slower than the AMP + ATP to ADP interconversion (*k*_cat_ = 330 ± 11 s^–1^ in the presence of Mg^2+^).^16^ The addition of magnesium increased the AP4A degradation rate only by a factor of 1.7, which is a small difference compared with the more than 44 000-fold increase reported for the ATP + AMP to ADP interconversion.^16^

**Figure 3:**
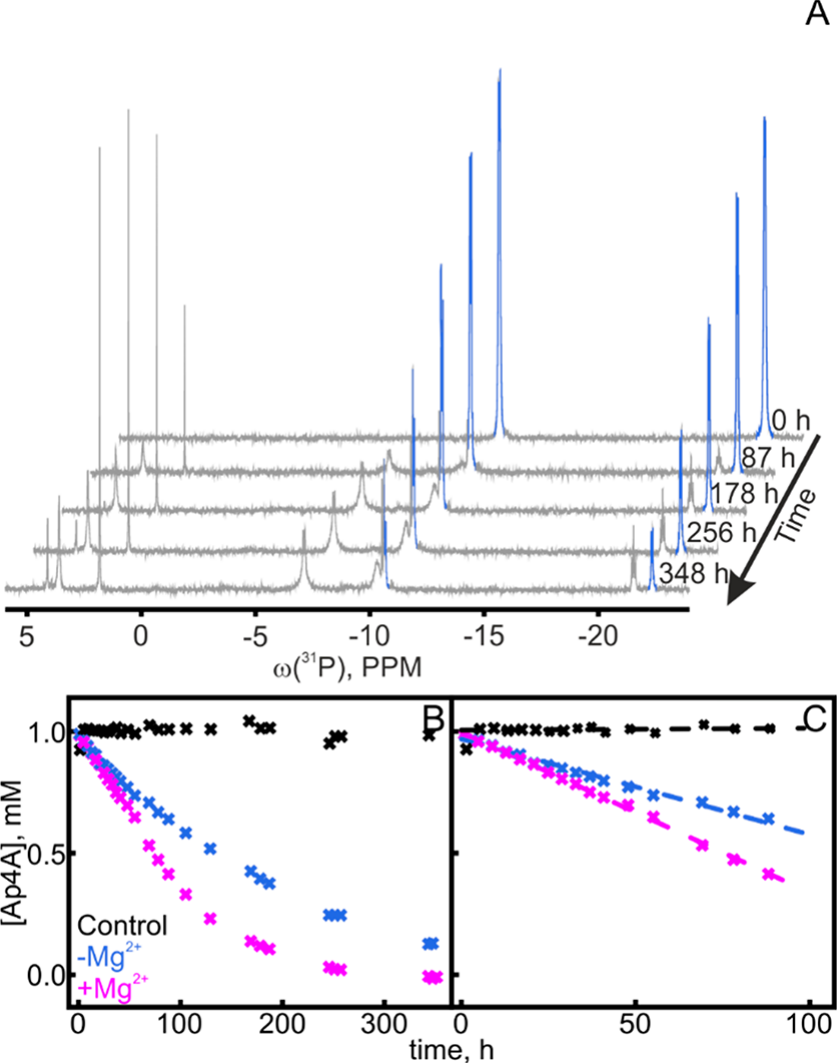
Hydrolysis of AP4A by AdK in the presence/absence of Mg^2+^ ions. (A) ^31^P NMR spectra of 1 mM AP4A at different time points from the addition of 200 μM wild-type AdK show a decrease of AP4A peaks (blue; ^31^P chemical shifts AP4A_α,α′_ = −11 ppm, AP4A_β,β′_ = −23 ppm), but an increase of all other peaks, e.g., for AMP (+4 ppm), free phosphate (+2 ppm), ADP_β_ (−6 ppm), and ATP_β_ (−21 ppm). Full assignment is shown in Figure S4. (B) The AP4A concentration was determined from peak area integrals in the absence (blue) and presence of 2 mM MgCl_2_ (pink) and as control without the enzyme (black). The first 100 h is enlarged in (C), where dashed lines represent linear fits to these first 100 h, thereby indicating an initial decay velocity of 6.6 μM AP4A/h with Mg^2+^ and 3.9 μM AP4A/h without Mg^2+^. One replicate (*n* = 1).

The slow AP4A hydrolysis by *E*. *coli* AdK suggests that it is likely a side reaction so far not characterized. However, it is an unexpected reaction, as *E*. *coli* AdK does not possess the amino acid sequence motifs that are found in known AP4A degrading enzymes, i.e., the Nudix sequence^21^ or the histidine triad.^22^

Many proteins with multitasking abilities have been identified.^23^ One subgroup, the so-called “moonlighting proteins,” performs different, often unrelated functions within one polypeptide chain,^23^ and AdK may belong to this.

AP4A binding has been shown to lead to a partitioning between open and closed states for a hyperthermophilic adenylate kinase using single-molecule experiments with optical tweezers.^24^ Here, we set out to assess the conformational changes of AdK induced by AP4A binding by EPR and NMR spectroscopy in solution (and not tethered to DNA as for the optical tweezers).

For the EPR experiments, two nitroxide (2,2,5,5-tetramethyl-1-pyrrolidinyloxy) spin labels were attached to an engineered AdK variant that contained amino acid replacements for labeling. One label was grafted at Lys50 in the AMPbd, and the other was grafted at Val148 in the ATPlid (Scheme S1). The sites were chosen on the basis of literature reporting the use of similar labeling positions for FRET and PRE measurements in an AdK from *Aquifex aeolicus*(25) and *E*. *coli*,^26^ in silico labeling, and distance simulations^27^ on apo and AP5A-bound AdK. Labeling yields were 60–150%, and structural and functional effects of the labels were negligible (Figures S5–S7, Table S3). The distance between the ATPlid and AMPbd was determined by four-pulse double electron–electron resonance (DEER) measurements.^28^ We compared the resulting distances for AP4A-bound AdK with the distances of AP5A-bound and substrate-free (apo) AdK (Figure 4A–C). The mean distance between the labels for the AP4A complex was longer (4.2 nm, Figure 4C, blue) than the equivalent distance of the AP5A complex (3.6 nm, red) and shorter than that of the substrate-free apo state (4.5 nm, gray). We estimated the statistical weights (populations) of open versus closed enzymes in the AP4A-bound conformation by fitting two Gaussians, one representing the open (apo) state and one the closed (AP5A-bound) state, to the data. This resulted in 58 ± 2% open and 42 ± 2% closed enzymes for the AP4A-bound conformation (Figures S8–S12, Table S4).

**Figure 4:**
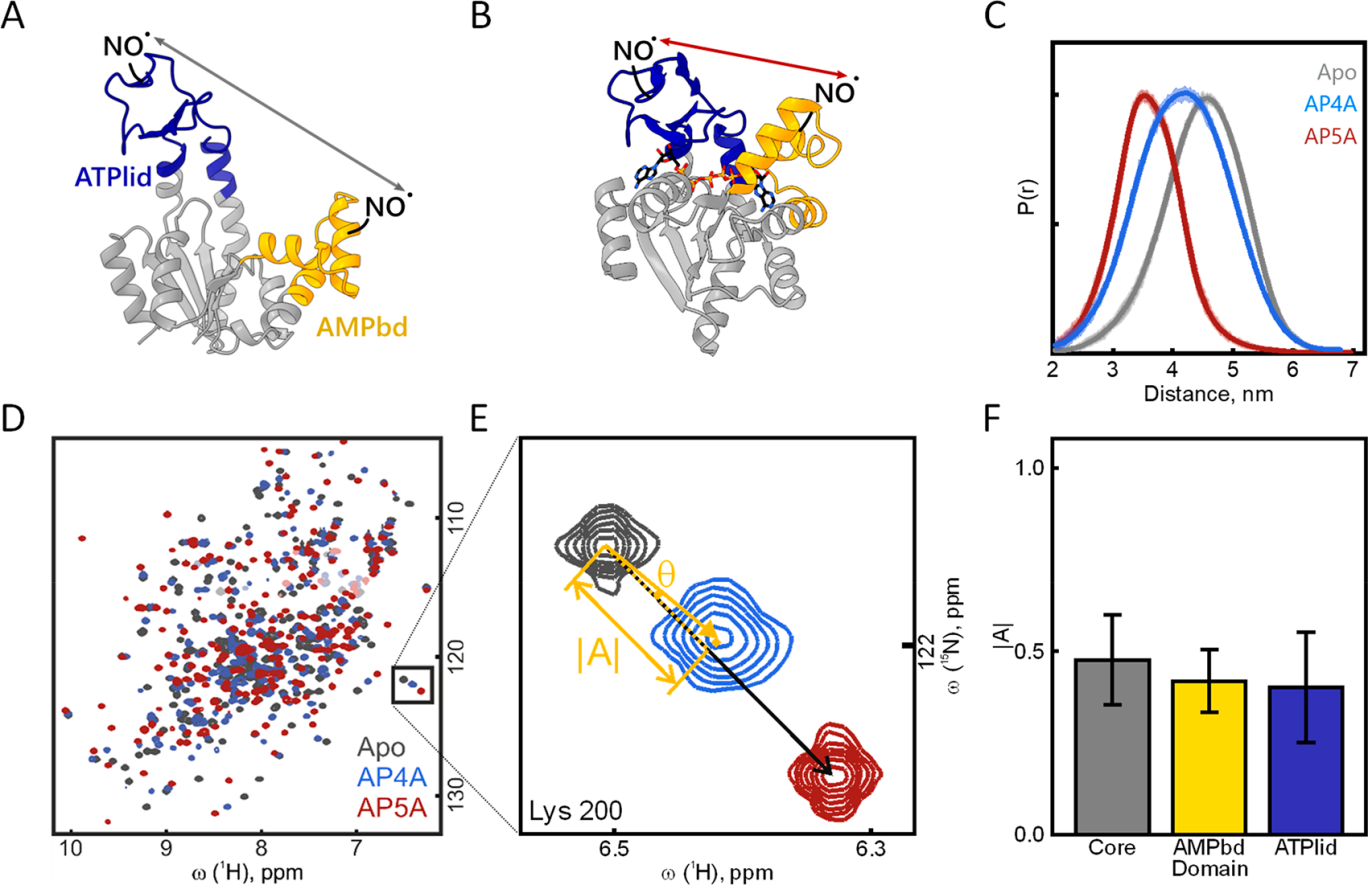
Dynamic binding of AP4A to AdK. (A) *E*. *coli* AdK apo conformation (PDB: 4AKE(11)) and (B) AP5A-bound conformation (AP5A shown as sticks, PDB: 1AKE(7)). The distances (arrows) between both nitroxide (NO•) labels in the AMPbd (yellow) and ATPlid (blue) were quantified by EPR spectroscopy. (C) Resulting distance distribution P(r) for 20 μM AdK (apo, gray) and with 100 μM AP5A (red) or 10 mM AP4A (blue). (D) ^1^H–^15^N HSQC NMR spectra of 200 μM wild-type AdK (apo, gray) with 1 mM AP5A (red) or 1 mM AP4A (blue). (E) Projection analysis exemplified for Lys200: the projection angle (θ) and the absolute value of the activation vector (|A|) are extracted for all individual residues. (F) The average |A| for all residues of the three domains is indicated with error bars for the standard deviation. Similar chemical shift changes over the protein suggest the same binding mode for AP4A as for AP5A.

Thus, AP4A-bound AdK is in a structural ensemble with both open and closed states populated with roughly equal weights. Consequently, AP4A binding to AdK is distinct to that of AP5A, which is strongly biased toward the closed state.^12,18^

To gain insight into the level of single amino acid residues, we turned to solution-state NMR spectroscopy. Inspection of ^1^H–^15^N HSQC spectra showed that the chemical shifts of AP4A in complex with AdK were roughly in between those of apo and AP5A-bound states (Figure 4D–F). Since AP4A binding occurs in the limit of fast exchange (Figure S13, i.e., the observed chemical shifts are population-weighted averages from the open and closed structural states^29^), a global quantification of chemical shift changes by means of a projection analysis^30^ was possible. The chemical shift vectors induced by AP4A were compared with those of AP5A as a reference for a fully closed state^18^ (Figures 4, S14, and S15). The residues were clustered according to the core, ATPlid, and AMbd domains. As for Lys200 (Figure 4E), amino acids from all three domains are described by an AP4A binding mechanism that involves roughly equal weights of open and closed states. On average over all residues, the fraction of closed state was 44 ± 13% for AP4A-bound AdK (Figure 4F, S15), which confirms the EPR results.

In conclusion, AdK binds to AP4A with both substrate-binding domains interconverting dynamically between the open and closed states. The time scale of the exchange is in the millisecond regime (or faster), as evidenced by the fast chemical shift exchange.

Thus, compared with the (nearly) fully closed AdK enzymes when bound to AP5A, we find approximately 50% of the AdK molecules open in the presence of AP4A. This suggests equal lifetimes for open and closed enzymes during AP4A hydrolysis. We think that both the AMPbd and the ATPlid close when binding AP4A, which then leads to AP4A hydrolysis (details in the Supporting Information section 3.6.3). It is also possible that the dynamic binding mode with the ATPlid partially detached from the core leaves AP4A “susceptible” to AdK-mediated hydrolysis. The latter is in line with the finding that an AdK variant failing to close over ATP has a sizable rate constant for ATP hydrolysis.^31^

In summary, we set out to characterize the structure of AP4A-bound adenylate kinase. Instead of locking the enzyme in a closed conformation (similar to AP5A), AP4A binding leads to approximately 50% open and 50% closed enzymes. Thus, AP4A stimulates an opening of the enzyme, which contrasts the AP5A case.^7,24^ It is relevant to mention that AP4A has the equivalent number of phosphate groups as the transition state of AdK in the ATP + AMP to ADP interconversion reaction. MD simulations suggest that the catalytic reaction is coupled to the large-scale collective conformational dynamics in AdK^16,32^ and the experimental finding that AP4A stimulates opening of AdK supports this coupling. We envision that the strain inferred by binding to AP4A (that is, minutely shorter than the transition-state compound) is the driver for enzyme opening. At the same time, AdK catalyzes the hydrolysis of AP4A, and it is likely because of the slow reaction rate that AP4A hydrolysis has been overseen for AdK.

In light of the intracellular concentrations of AdK’s ligands, which are very high for ATP (1–10 mM in *E*. *coli*)^33,34^ and in the lower μM range for AP4A,^1,2^ a real competition of these substrates is unlikely, and the intracellular effects or AdK-mediated AP4A hydrolysis remain to be established. However, it is interesting to speculate about the potential ramification of AP4A binding to AdK. One potential consequence of AdK binding to AP4A is the downregulation of AdK, which, in turn, would lead to a shifted balance between AMP, ADP, and ATP with an impact on the fitness of *E*. *coli*. Second, the slow AP4A hydrolysis by AdK observed here in vitro might be faster in vivo. In case AP4A is, indeed, a metabolic side-product, this would suggest recycling of AP4A by AdK. It is known that certain forms of AdK in mammalian cells^35,36^ are vital in the response to oxidative stress, the form of stress that causes the highest increase in AP4A. The binding and degradation of AP4A by AdK might be a stress response and, hence, improve the *E*. *coli*’s fitness during oxidative stress. Still, AP4A remains enigmatic, and its intracellular effects on AdK need to be studied further.

## Supporting Information Available

Experimental procedures and additional iTC, crystallography, enzymatic assay, CD, NMR, and EPR spectroscopy results (PDF)

## Accession Codes

AdK: UniProtKB P69441. Protein data bank entries: 8CRG.

## Abbreviations

AMP: adenosine 5′-monophosphate
ADP: adenosine 5′-diphosphate
ATP: adenosine 5′-triphosphate
HSQC: heteronuclear single quantum coherence spectroscopy

## References

[ref1] Ferguson F.; McLennan A. G.; Urbaniak M. D.; Jones N. J.; Copeland N. A. Re-evaluation of diadenosine tetraphosphate (Ap4A) from a stress metabolite to bona fide secondary messenger. Front. Mol. Biosci. 7 2020, 7, 606807 10.3389/fmolb.2020.606807. PMC7705103 33282915

[ref2] Despotović D.; Brandis A.; Savidor A.; Levin Y.; Fumagalli L.; Tawfik D. S. Diadenosine tetraphosphate (Ap4A) – an *E. coli* alarmone or a damage metabolite?. FEBS J. 2017, 284, 2194–2215. 10.1111/febs.14113. 28516732

[ref3] Götz K. H.; Mex M.; Stuber K.; Offensperger F.; Scheffner M.; Marx A. Formation of the alarmones diadenosine triphosphate and tetraphosphate by ubiquitin- and ubiquitin-like-activating enzymes. Cell Chem. Biol. 26 2019, 26, 1535–1543. 10.1016/j.chembiol.2019.08.004. 31492597

[ref4] Fraga H.; Fontes R. Enzymatic synthesis of mono and dinucleoside polyphosphates. Biochim. Biophys. Acta - Gen. Subj. 2011, 1810, 1195–1204. 10.1016/j.bbagen.2011.09.010. 21978831

[ref5] Jankowski V.; van der Giet M.; Mischak H.; Morgan M.; Zidek W.; Jankowski J. Dinucleoside polyphosphates: strong endogenous agonists of the purinergic system. Br. J. Pharmacol. 2009, 157, 1142–1153. 10.1111/j.1476-5381.2009.00337.x. 19563527 PMC2743832

[ref6] Luciano D. J.; Belasco J. G. Np4A alarmones function in bacteria as precursors to RNA caps. Proc. Natl. Acad. Sci. U. S. A. 2020, 117, 3560–3567. 10.1073/pnas.1914229117. 32019889 PMC7035476

[ref7] Müller C. W.; Schulz G. E. Structure of the complex between adenylate kinase from *Escherichia coli* and the inhibitor Ap5A refined at 1.9 Å resolution. A model for a catalytic transition state. J. Mol. Biol. 1992, 224, 159–177. 10.1016/0022-2836(92)90582-5. 1548697

[ref8] Reinstein J.; Vetter R. I.; Schlichting I.; Rösch P.; Wittinghofer A.; Goody S. R. Fluorescence and NMR investigations on the ligand binding properties of adenylate kinases. Biochemistry 1990, 29, 7440–7450. 10.1021/bi00484a013. 2223775

[ref9] Verma A.; Åberg-Zingmark E.; Sparrman T.; Ul Mushtaq A.; Rogne P.; Grundström C.; Berntsson R.; Sauer U. H.; Backman L.; Nam K.; Sauer-Eriksson E.; Wolf-Watz M. Insights into the evolution of enzymatic specificity and catalysis: From *Asgard* archaea to human adenylate kinases. Sci. Adv. 2022, 8, 4089 10.1126/sciadv.abm4089. PMC9635829 36332013

[ref10] Kerns S. J.; Agafonov R. V.; Cho Y.-J. J.; Pontiggia F.; Otten R.; Pachov D. V.; Kutter S.; Phung L. A.; Murphy P. N.; Thai V.; Alber T.; Hagan M. F.; Kern D. The energy landscape of adenylate kinase during catalysis. Nat. Struct. Mol. Biol. 2015, 22, 124–131. 10.1038/nsmb.2941. 25580578 PMC4318763

[ref11] Müller C. W.; Schlauderer G. J.; Reinstein J.; Schulz G. E. Adenylate kinase motions during catalysis: an energetic counterweight balancing substrate binding. Structure 1996, 4, 147–156. 10.1016/S0969-2126(96)00018-4. 8805521

[ref12] Henzler-Wildman K. A.; Thai V.; Lei M.; Ott M.; Wolf-Watz M.; Fenn T.; Pozharski E.; Wilson M. A.; Petsko G. A.; Karplus M.; Hübner C. G.; Kern D. Intrinsic motions along an enzymatic reaction trajectory. Nature 2007, 450, 838–843. 10.1038/nature06410. 18026086

[ref13] Lu J.; Scheerer D.; Haran G.; Li W.; Wang W. Role of repeated conformational transitions in substrate binding of adenylate kinase. J. Phys. Chem. B 2022, 126, 8188–8201. 10.1021/acs.jpcb.2c05497. 36222098 PMC9589722

[ref14] Kovermann M.; Grundström C.; Sauer-Eriksson A. E.; Sauer U. H.; Wolf-Watz M. Structural basis for ligand binding to an enzyme by a conformational selection pathway. Proc. Natl. Acad. Sci. U.S.A. 2017, 114, 6298–6303. 10.1073/pnas.1700919114. 28559350 PMC5474765

[ref15] Hanson J. A.; Duderstadt K.; Watkins L. P.; Bhattacharyya S.; Brokaw J.; Chu J.-W. W.; Yang H. Illuminating the mechanistic roles of enzyme conformational dynamics. Proc. Natl. Acad. Sci. U. S. A. 2007, 104, 18055–18060. 10.1073/pnas.0708600104. 17989222 PMC2084295

[ref16] Ojeda-May P.; Mushtaq A. U. I.; Rogne P.; Verma A.; Ovchinnikov V.; Grundström C.; Dulko-Smith B.; Sauer U. H.; Wolf-Watz M.; Nam K. Dynamic connection between enzymatic catalysis and collective protein motions. Biochemistry 2021, 60, 2246–2258. 10.1021/acs.biochem.1c00221. 34250801 PMC8297476

[ref17] Stiller J. B.; Otten R.; Häussinger D.; Rieder P. S.; Theobald D. L.; Kern D. Structure determination of high-energy states in a dynamic protein ensemble. Nature 2022, 603, 528–535. 10.1038/s41586-022-04468-9. 35236984 PMC9126080

[ref18] Ådén J.; Wolf-Watz M. NMR identification of transient complexes critical to adenylate kinase catalysis. J. Am. Chem. Soc. 2007, 129, 14003–14012. 10.1021/ja075055g. 17935333

[ref19] Orädd F.; Ravishankar H.; Goodman J.; Rogne P.; Backman L.; Duelli A.; Pedersen M. N.; Levantino M.; Wulff M.; Wolf-Watz M.; Andersson M. Tracking the ATP-binding response in adenylate kinase in real time. Sci. Adv. 2021, 7, 5514 10.1126/sciadv.abi5514. PMC8597995 34788091

[ref20] Rogne P.; Andersson D.; Grundström C.; Sauer-Eriksson E.; Linusson A.; Wolf-Watz M. Nucleation of an activating conformational change by a cation-π interaction. Biochemistry 2019, 58, 3408–3412. 10.1021/acs.biochem.9b00538. 31339702

[ref21] Ge H.; Chen X.; Yang W.; Niu L.; Teng M. Crystal structure of wild-type and mutant human Ap4A hydrolase. Biochem. Biophys. Res. Commun. 2013, 432, 16–21. 10.1016/j.bbrc.2013.01.095. 23384440 PMC7092880

[ref22] Hou W. T.; Li W. Z.; Chen Y.; Jiang Y. L.; Zhou C. Z. Structures of yeast Apa2 reveal catalytic insights into a canonical Ap4A phosphorylase of the histidine triad superfamily. J. Mol. Biol. 2013, 425, 2687–2698. 10.1016/j.jmb.2013.04.018. 23628156

[ref23] Huberts D. H. E. W.; van der Klei I. J. Moonlighting proteins: An intriguing mode of multitasking. Biochim. Biophys. Acta - Mol. Cell Res. 2010, 1803, 520–525. 10.1016/j.bbamcr.2010.01.022. 20144902

[ref24] Pelz B.; Žoldák G.; Zeller F.; Zacharias M.; Rief M. Subnanometre enzyme mechanics probed by single-molecule force spectroscopy. Nat. Commun. 2016, 7, 10848 10.1038/ncomms10848. 26906294 PMC4770092

[ref25] Henzler-Wildman K. A.; Thai V.; Lei M.; Ott M.; Wolf-Watz M.; Fenn T.; Pozharski E.; Wilson M. A.; Petsko G. A.; Karplus M.; Hübner C. G.; Kern D. Intrinsic motions along an enzymatic reaction trajectory. Nature 2007, 450, 838–844. 10.1038/nature06410. 18026086

[ref26] Rogne P.; Rosselin M.; Grundström C.; Hedberg C.; Sauer U. H.; Wolf-Watz M. Molecular mechanism of ATP versus GTP selectivity of adenylate kinase. Proc. Natl. Acad. Sci. U.S.A. 2018, 115, 3012–3017. 10.1073/pnas.1721508115. 29507216 PMC5866598

[ref27] Jeschke G. MMM: A toolbox for integrative structure modeling. Protein Sci. 2018, 27, 76–85. 10.1002/pro.3269. 28799219 PMC5734387

[ref28] Jeschke G. DEER distance measurements on proteins. Annu. Rev. Phys. Chem. 2012, 63, 419–46. 10.1146/annurev-physchem-032511-143716. 22404592

[ref29] Kleckner I. R.; Foster M. P. An introduction to NMR-based approaches for measuring protein dynamics. Biochim. Biophys. Acta - Proteins Proteomics 2011, 1814, 942–968. 10.1016/j.bbapap.2010.10.012. PMC3061256 21059410

[ref30] Selvaratnam R.; Vanschouwen B.; Fogolari F.; Mazhab-Jafari M. T.; Das R.; Melacini G. The projection analysis of NMR chemical shifts reveals extended EPAC autoinhibition determinants. Biophys. J. 2012, 102, 630–639. 10.1016/j.bpj.2011.12.030. 22325287 PMC3274826

[ref31] Ådén J.; Weise C. F.; Brännström K.; Olofsson A.; Wolf-Watz M. Structural topology and activation of an initial adenylate kinase-substrate complex. Biochemistry 2013, 52, 1055–1061. 10.1021/bi301460k. 23339454

[ref32] Dulko-Smith B.; Ojeda-May P.; Ådén J.; Wolf-Watz M.; Nam K. Mechanistic basis for a connection between the catalytic step and slow opening dynamics of Adenylate Kinase. J. Chem. Inf. Model. 2023, 63, 1556–1569. 10.1021/acs.jcim.2c01629. 36802243 PMC11779523

[ref33] Yaginuma H.; Kawai S.; Tabata K. V.; Tomiyama K.; Kakizuka A.; Komatsuzaki T.; Noji H.; Imamura H. Diversity in ATP concentrations in a single bacterial cell population revealed by quantitative single-cell imaging. Sci. Rep. 2014, 4, 6522 10.1038/srep06522. 25283467 PMC4185378

[ref34] Bennett B. D.; Kimball E. H.; Gao M.; Osterhout R.; Van Dien S. J.; Rabinowitz J. D. Absolute metabolite concentrations and implied enzyme active site occupancy in *Escherichia coli*. Nat. Chem. Biol. 2009 58 2009, 5, 593–599. 10.1038/nchembio.186. PMC2754216 19561621

[ref35] Liu R.; Ström A. L.; Zhai J.; Gal J.; Bao S.; Gong W.; Zhu H. Enzymatically inactive adenylate kinase 4 interacts with mitochondrial ADP/ATP translocase. Int. J. Biochem. Cell Biol. 2009, 41, 1371–1380. 10.1016/j.biocel.2008.12.002. 19130895 PMC2676352

[ref36] Fujisawa K. Regulation of adenine nucleotide metabolism by adenylate kinase isozymes: physiological roles and diseases. Int. J. Mol. Sci. 2023, 24, 5561 10.3390/ijms24065561. 36982634 PMC10056885

