# Supplementary material for: Insights into Enzymatic Catalysis from Binding and Hydrolysis of Diadenosine Tetraphosphate by E. coli Adenylate Kinase
Source: Biochemistry. 2023 Jul 7;62(15):2238–43. doi: 10.1021/acs.biochem.3c00189 (PMC10399197; doi:10.1021/acs.biochem.3c00189)
Supplement: Supplementary file 1 — bi3c00189_si_001.pdf [file bi3c00189_si_001.pdf]

Supporting Information for Insights into enzymatic catalysis from binding and hydrolysis of diadenosine-tetraphosphate by *E. coli* adenylate kinase

Sonja Tischlik[1], *Melanie Oelker*[2], *Per Rogne*[2], *A. Elisabeth Sauer-Eriksson*[2, 3], *Malte Drescher*[1] *and Magnus Wolf-Watz**[2, 3]

1 Department of Chemistry, Konstanz Research School Chemical Biology, University of Konstanz, 78464 Konstanz, Germany

2 Department of Chemistry, Umeå University, SE-901 87 Umeå, Sweden

3 Centre of Microbial Research (UCMR), Umeå University, SE-901 87 Umeå, Sweden

* To whom correspondence may be addressed: magnus.wolf-watz@umu.se

CONTENT

1 Chemicals 3
2 Methods 3
2.1 Protein preparation 3
2.1.1 Cloning 3
2.1.2 Protein expression and purification 3
2.2 Isothermal Titration Calorimetry (ITC) 4
2.3 Crystal data collection and refinement statistics 4
2.3.1 Crystallization 4
2.3.2 Data collection 4
2.3.3 Processing, phasing and refinement 4
2.4 Circular Dichroism (CD) 5
2.5 Enzymatic assay 5
2.6 NMR 6
2.6.1 NMR sample preparation 6
2.6.2 ^{31}P NMR 6
2.6.3 ^{1}H ^{15}N HSQC spectra 6
2.6.4 Projection analysis 6
2.7 Site-directed spin labeling for EPR experiments 7
2.7.1 CuAAC 7
2.7.2 Cysteine-maleimide coupling 8
2.8 EPR 8
2.8.1 Continuous-wave (cw) EPR 8
2.8.2 Pulsed EPR 8
3 Supplementary Data 11
3.1 iTC data 11
3.2 Crystallography data 12
3.3 ^{31}P NMR data 16
3.4 CD data 17
3.5 Enzymatic assay & in-silico labelling 18
3.6 EPR 19
3.6.1 Labelling controls 19
3.6.2 Quantification of the lid-to-lid EPR distance measurements 20
3.6.3 AP4A binding mode 21
3.6.4 Controls for reproducibility 23
3.7 ^{1}H ^{15}N HSQC NMR 26
4 Abbreviations 29
5 References 30

1 CHEMICALS

All adenosine nucleotides were purchased from Merck and used without any further purification. The non-canonical amino acid *para*-propargyloxy-L-phenylalanine (pPfF) was purchased from Iris Biotech GmbH. Azido-PROXYL was synthesized according to published procedures[1].

2 METHODS

2.1 PROTEIN PREPARATION

2.1.1 Cloning

Wild-type *E. coli* adenylate kinase (AdK) was overexpressed from a self-inducing plasmid (pEAK91) using previously published procedures[2], as well as the Val148Cys mutant used for PRE-NMR measurements[3]. For EPR measurements, a K50TAG was introduced as additional mutation for double-labeling and subsequent EPR distance measurements. The amber-stop-codon (TAG) enabled the insertion of the non-canonical amino acid *para*-propargyloxy-L-phenylalanine (pPrF)[4] for subsequent labeling over Copper(I)-catalyzed azido-alkyne click reaction (CuAAC)[5]. The used primers are detailed in Table S1.

Table S1: Primer sequences used for K50TAG mutations in *E. coli* AdK.

E. coli AdK mutation	*Primer sequence*
K50TAG fwd (3' -> 5')	***CCGAGCTGGGTAAACAAGCATAGGACATTATGGATGCTGGC***
K50TAG rev (5' -> 3')	***AGCATCCATAATGTCCTATGCTTGTTTACCCAGCTCGGAGCC***

2.1.2 Protein expression and purification

Chemically competent BL21-gold (DE3) *E. coli* cells were transformed with the plasmid for *E. coli* AdK wild type according to published procedures[2,3]. The double mutant Lys50TAG/Val148Cys plasmid was co-transformed with an additional plasmid for the aminoacetyl-tRNA-syntethase/tRNA pair (pEVOL_pCNPhe provided by the Schultz lab[6]) that can incorporate the non-canonical amino acid pPrF at the position of the amber stop codon[5].

To obtain a uniformly ^{15}N-labeled enzyme for the ^{15}N-edited NMR experiments, expression cells were grown in minimal medium (M9) with $^{15}NH_4Cl$ as the sole nitrogen source. Otherwise, LB-medium was used.

The expressed proteins were purified in two steps using first affinity chromatography over a Blue sepharose column (26 ml column volume) using 50 mM Tris-buffer with 1 M NaCl, pH 7.5 for elution, followed by gel filtration as second step. The protein concentration was determined via absorption measurement at 280 nm on a BioPhotometer (Eppendorf). Purified protein was stored in 30 mM MOPS, 50 mM NaCl buffer at pH 7.0, which was supplemented with 1 mM TCEP for the cysteine mutants to inhibit the formation of dimers through disulfide bond formation.

2.2 Isothermal Titration Calorimetry (ITC)

To determine the binding affinity of AdK to the AP4A ligand, ITC was carried out with wild-type AdK at a MicroCal ITC200 at 298 K. A 1.15 mM AP4A solution was titrated in 26 steps to a 168 µM solution of wild type AdK. As buffer, 30 mM MOPS, 50 mM NaCl, pH 7.0 was used. The dissociation constant K_d, binding enthalpy ΔH^0, binding entropy $-T\Delta S^0$ and the binding ratio (N) were determined using the analysis program provided by the ITC manufacturer. The results are shown in Figure S1.

2.3 Crystal data collection and refinement statistics

2.3.1 Crystallization

For co-crystallization of AdK with AP4A, 23.3 mg/mL (980 µM) of AdK in 30 mM MOPS, 50 mM NaCl buffer at pH 7.0 was mixed with 4 µl 130 mM AP4A resulting in a protein to ligand ratio of 1:10.6 and a final concentration of AP4A of 0.9 mM. Crystallization was performed by sitting drop vapor diffusion method at 291 K. The AdK-AP4A mixture was mixed in a 1:1 ratio (0.5 µL + 0.5 µL) with the crystallization solution containing 20 % (w/v) PEG 3350 and 100 mM Bis-tris propane at pH 6.5. Grown crystals were flash-frozen in liquid nitrogen after a short soaking in a cryoprotection solution, consisting of the corresponding crystallization condition with the PEG 3350 concentration increased to 35 % (w/v). Crystals appeared after 3 - 8 days and were mounted after 1 - 3 weeks. It is noteworthy that this is in the same time-scale as the described AP4A hydrolysis.

2.3.2 Data collection

Data collection was performed at 100 K at beamline BioMAX at MAX IV (Lund, Sweden)[7].

We acknowledge MAX IV Laboratory for time on Beamline BioMAX under Proposal 20210468. Research conducted at MAX IV, a Swedish national user facility, is supported by the Swedish Research council under contract 2018-07152, the Swedish Governmental Agency for Innovation Systems under contract 2018-04969, and Formas under contract 2019-02496.

2.3.3 Processing, phasing and refinement

The dataset was processed with the XDS program package[8]. For the subsequent steps, several programs from the CCP4[9] and Phenix[10] software suites were used. The structure was solved by molecular replacement with the program MOLREP[11] using a (unpublished) structure from a previously recorded dataset. The structure was once refined by a rigid body refinement as implemented in REFMAC5[12] and a simulated annealing refinement as implemented in phenix.refine[13] to reduce model bias. Further refinement was performed by several cycles of manual model correction in *Coot*[14] and automated refinement by either REFMAC5[12] or phenix.refine[13]. The final structure was deposited in the Protein Data Bank (PDB) with the PDB ID: 8CRG. Coordinates and restraints for the ligand AP4A were generated with AceDRG[15] as implemented in the ligand builder of *Coot*[14]. Figures were generated with PyMOL[16]. Electron density maps for figures were generated with phenix.mtz2map.

2.4 CIRCULAR DICHROISM (CD)

To check whether spin-labeling affect the secondary structure of the AdK, CD spectra of wild-type protein, mutant and labeled mutant were recorded (Figure S5). The protein samples were diluted to 25 µM in 30 mM MOPS, 50 mM NaCl, pH 7.0 and 80 µL were transferred to 0.5 mm demountable cuvettes. Spectra were recorded on a JASCO J-715 spectropolarimeter by continuously scanning from 280 to 180 nm with a scanning speed of 50 nm/min, response time of 4 s and a bandwidth of 1.0 nm at 293 K. The recorded spectra were averaged over 10 scans, baseline- and background-corrected with the same buffer. For the conversion of the spectrometer unit (CD-signal in mdeg) to the molar residue ellipticity (MRE), the following formula was used:

$$MRE = [M/N_{aa} \cdot CD\text{-}signal/1000]/(10 \cdot d \cdot c)$$

With the molar mass M in g/mol, N_{aa} as the number of amino acid residues in the protein, c in g/L, and d = 0.05 cm. The results are shown in Figure S5.

2.5 ENZYMATIC ASSAY

To assure that the AdK is still active when 2 spin labels are attached to the catalytically important ATP- and AMPbd, an enzymatic assay was performed. The Michaelis constant K_M and the catalytic turn-over rate k_{cat} were determined with an enzymatic assay that couples the generation of ADP by the adenylate kinase to the (fast) reaction of the two enzymes pyruvate kinase and NADPH dehydrogenase[17,18]. In its course, NADH, H^+ is oxidized to NAD^+ which can be followed by UV/Vis spectroscopy because of the strong absorbance from NADH at 340 nm (ε_{340nm}(NADH) = 6300 $M^{-1}cm^{-1}$ in H_2O, T = 298 K). From the consumption of NADH, the formation of ADP by the AdK can be deduced, allowing to fit the reaction rates (normalized to enzyme concentration; v/[AdK]) to the Michaelis-Menten equation[17,18]:

$$\frac{\boldsymbol{v}}{[\boldsymbol{AdK}]}([\boldsymbol{ATP}]) = \frac{\boldsymbol{k_{cat}} \cdot [\boldsymbol{S}]}{\boldsymbol{K_M} + [\boldsymbol{S}]} \quad \textbf{(1)}$$

Where v/[AdK]([ATP]) is the normalized reaction rate at the ATP concentration, k_{cat} is the catalytic turnover number and K_M is the Michaelis constant.

2.6 NMR

2.6.1 NMR sample preparation

For NMR experiments, 500 µl of 200 µM AdK (with or without ligands) in 30 mM MOPS pH 7.0 supplemented with 50 mM NaCl were used. 7 % D_2O was added to provide the field-frequency lock signal of the spectrometer.

2.6.2 ^{31}P NMR

In order to determine the breakdown rate of AP4A, a sample containing 1 mM AP4A and 200 µM of wild-type AdK in a 30 mM MOPS, 50 mM NaCl buffer at pH 7.0 was used. When measuring in the presence of Mg^{2+}, the sample was supplemented with 2 mM of $MgCl_2$. The breakdown of AP4A into ATP, ADP, AMP, and inorganic phosphorous was then monitored by the recording of one-dimensional ^{31}P NMR spectra at predetermined time intervals. Both spectra recording and incubation between the spectra were done at 295 K. The NMR spectra were recorded on a 600 MHz Bruker Advance III spectrometer (^{31}P frequency: 243 MHz) equipped with a 5 mm broadband cryo probe. The sweep width was 60 ppm (16384 Hz) and 256 scans were recorded for each spectrum. The concentration of each phosphorous species was determined by integration of each peak using Topspin 3.6.1 (Bruker Biospin), comparing it to the total integral under all peaks.

2.6.3 ^{1}H ^{15}N HSQC spectra

^{1}H ^{15}N heteronucelar single quantum coherence (HSQC) spectra were recorded to monitor the overall protein structure and to detect possible local changes upon ligand addition. The spectra of 200 µM AdK in 30 mM MOPS, 50 mM NaCl buffer at pH 7.0 buffer were recorded on an 850 MHz Bruker Avance III spectrometer equipped with a 5 mm HCN cryoprobe with 1024*256 points over a 13*30 ppm sweep width with 8 scans used for each line. The spectra were processed using NmrPipe[19] and the peaks were picked using Ansig[20] or Sparky[21,22]. The assignments were based on previously published values for the apo form[23]. The k_D was determined, based on induced chemical shifts in both the ^{1}H and the ^{15}N dimension, by fitting the one-site binding equation[24] to the determined chemical shift using in house developed MatLab (Mathworks) scripts.

2.6.4 Projection analysis

To quantify the structural changes induced by AP4A, a projection analysis of chemical shifts in the ^{1}H ^{15}N HSQC spectra of wild-type AdK was performed according to Selvaratnam and co-workers[25]. The projection analysis compares the vectors of chemical shift defined from the substrate free state to a ligand bound state. Two parameters are then extracted, the projection angle (θ) and the absolute value of the activation vector (|A|, main text, Fig. 4E, Figure S18A, B). The angle contains information of the similarity of the binding process for the two ligands that are compared, whereas the activation vector contains information on the statistical weight of the contributing states (here open and closed states). The spectrum of apo and AP5A saturated AdK was used as reference for the fully open and fully closed state, respectively. The projected apo-AP4A vector determines how much of the chemical shift induced by AP4A binding can be attributed to the same conformal change as AP5A binding. This procedure was performed for every assigned residue, but only residues where the angle θ between the apo-to-AP5A vector and apo-to-AP4A vector was smaller than 25.8 ° (= cos(0.9)) were taken into account[25] (Figure S18C, D). The average projected chemical shift induced by AP4A binding was 0.45 ± 0.13 of the chemical shifts induced by AP5A.

2.7 SITE-DIRECTED SPIN LABELING FOR EPR EXPERIMENTS

For EPR distance measurements, two paramagnetic labels are required to measure the distance between them. Here, one PROXYL label was introduced first at position Lys50 via Copper(I)-catalyzed azido-alkyne click reaction (CuAAC) and a second via cysteine-maleimide coupling at position Val148Cys (Scheme S1).

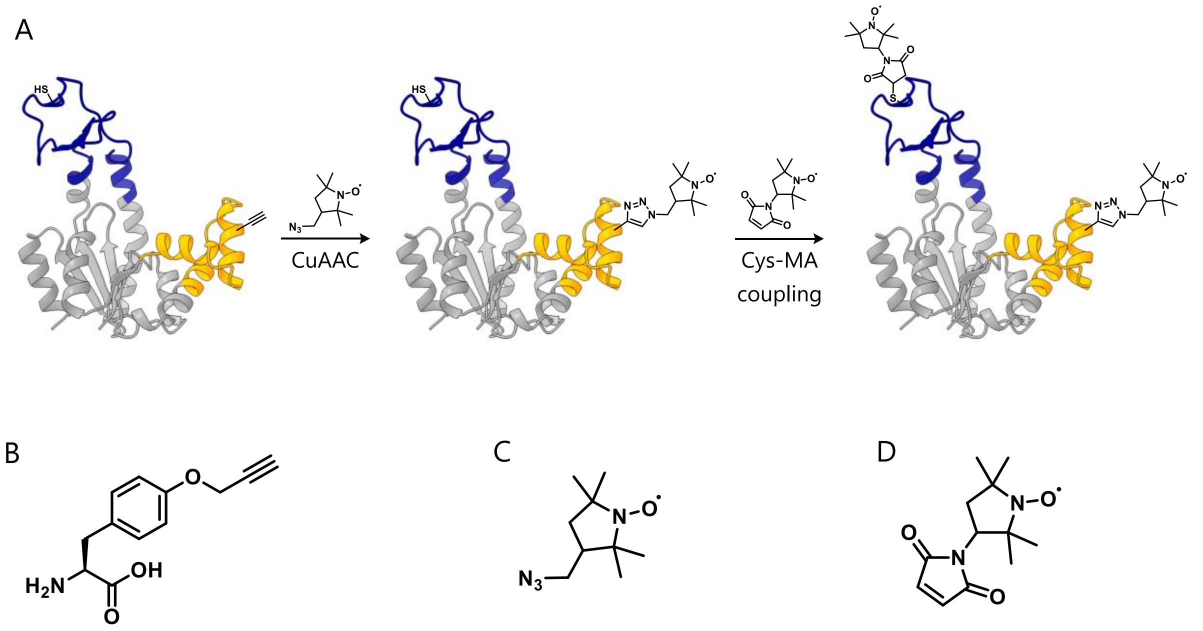

Scheme S1. Spin labeling strategy and chemical structures of the used non-canonical amino acid and spin labels.
(**A**) Selective labeling strategy of Lys50pPrF via CuAAC with azido-PROXYL and Val148Cys via Cys-MA coupling with maleimido-PROXYL. PDB ID: 4AKE[26]. Chemical structures of (**B**) the non-canonical amino acid *para*-propargyloxy-*L*-phenylalanine (pPrF) (**C**) azido-PROXYL and (**D**) maleimido-PROXYL.

2.7.1 CuAAC

Coupling of azido-PROXYL to the non-canonical amino acid pPrF was performed as described previously by Kucher et al.[1] As first step, the buffer was exchanged to PBS with 1 mM TCEP, pH 7.5, as labeling in 30 mM MOPS, 50 mM NaCl pH 7.0 was found to be less efficient. Then, copper(II)-sulphate ($CuSO_4$) and the ligand 2-(4-((bis((1-(tert-butyl)-1H-1,2,3-triazol-4-yl)methyl)amino)methyl)-1H-1,2,3-triazol-1-yl)acetic acid (BTTAA) were mixed in deionized water in a 1:3 ratio. Then, labeling reagents were diluted with PBS buffer pH 7.5, the protein and azido-PROXYL in DMSO (100 mM stock solution) were added. Ascorbic acid was added in a 1:1 ratio to reduce Cu(II) ions to the catalytically active Cu(I) species. The final concentrations were: 1 mM copper sulfate, 3 mM BTTAA, 30 – 50 µM protein, 1 mM spin label and 1 mM sodium ascorbate. The reaction was incubated for 40 min, 298 K and 400 rpm. After the reaction, a 5-fold excess of EDTA over copper was added to complex free Cu-ions. The free label and other reactants were removed by size exclusion chromatography using a Sephadex SEC 26/10 column (GE Healthcare).

2.7.2 Cysteine-maleimide coupling

As second labeling step, maleimido-PROXYL was coupled to the introduced cysteine at position Val148. The protein concentration was adjusted to 20 - 50 µM in PBS buffer, pH 7.5. A 5-fold excess was added in 2 steps: first, a 2.5-fold excess of maleimido-PROXYL was added for 30 min at 293 K, 300 rpm. Another 2.5-fold excess of maleimido-PROXYL was added for additional 30 min under the same conditions. The reaction was carried out in two steps to ensure low excess of label during the reaction to not promote labeling of the 2nd naturally occurring cysteine at position 77. Free label was removed by size exclusion chromatography using a Sephadex SEC 26/10 column (GE Healthcare).

To prove that no significant labeling at Cys77 occurs, 4p-DEER measurements on AdK spin labeled only with maleimido-PROXYL were performed (Figure S8). In case both Val148Cys and Cys77 positions were labeled with the above-described procedure, clear dipolar oscillations and modulation depth were expected. This was not the case (Figure S8), proving efficient labeling only at position Val148Cys.

Labeling and spin-label concentrations were monitored by cw EPR (section 2.8.1). The influence of the labels on secondary structure was examined by CD spectroscopy (section 2.4) and the influence on ATP binding and activity via an enzymatic assay (Enzymatic assay & in-silico labelling

Table S3).

2.8 EPR

2.8.1 Continuous-wave (cw) EPR

To determine the spin labeling efficiencies of labeled AdK, cw EPR spectra were recorded. The spectra were recorded at r.t. (293 – 297 K) on an EMX-Nano benchtop spectrometer (Bruker Biospin) at 9.4 GHz (X-band). Typically, 20 µL of the purified protein sample was filled into a glass capillary (HIRSCHMANN ringcaps®; inner diameter 1.02 mm). Spectra were recorded with a modulation amplitude of 0.8 G, 1 mW microwave power, a sweep width of 200 G and a sweep time of 80 s. To improve the signal-to-noise ratio, the spectra were averaged over 16 scans. Quantitative spin concentrations were obtained using the built-in EMXnano reference-free spin counting module (Xenon software, Bruker), from which the labeling efficiency, defined as the ratio of spin to protein concentration, was calculated.

2.8.2 Pulsed EPR

All pulsed EPR experiments were performed at 34 GHz (Q band) with a microwave attenuation of 0 dB. Echo signals were detected in integrator mode with a video bandwidth of 200 MHz. The shot repetition rate was set to 4080 µs.

2.8.2.1 Instrumentation

All pulsed experiments were performed on a Q-band Elexsys E580 spectrometer (Bruker Biospin). The device is equipped with a SpinJet-AWG unit (Bruker Biospin) and a 150 W pulsed TWT amplifier (Applied Systems Engineering), allowing the use of shaped microwave pulses. The temperature was maintained at 50 K with the EPR Flexline helium recirculation system (CE-FLEX-4K-0110, Bruker Biospin, ColdEdge Technologies), comprising a cold head (expander, SRDK-408D2) and a F-70H compressor (both SHI cryogenics), controlled by an Oxford Instruments Mercury ITC. For all pulsed EPR measurements, an overcoupled, commercial Q-band resonator (ER5106QT-2, Bruker Biospin) was used with a Q-value of approximately 200.

2.8.2.2 4p-DEER experiments

DEER samples were prepared by mixing the spin-labeled AdK with the respective ligands in deuterated 30 mM MOPS, 50 mM NaCl, pH = 7.0 and incubate it 15 min at room temperature. Then, 60 % of d8-glycerol was added as cryoprotectant and each sample with a total volume of 60 µl and a final concentration of 20 µM AdK was transferred to a quartz tube with 3 mm outer diameter (fused quartz tubing, Technical Glass Products). The samples were flash-frozen in liquid nitrogen and stored at 193 K.

4p-DEER measurements were recorded with the standard pulse sequence:

$$(\pi/2)_{obs} - \tau_1 - \pi_{obs} - t - t_{pump} - (\tau_1 + \tau_2 - t) - \pi_{obs} - \tau_2 - echo$$

With interpulse delays of τ_1 = 400 ns, τ_2 = 6 µs (or more). The pump pulse position was incremented in t = 8 ns steps. Nuclear modulation artifacts were suppressed by averaging 8 traces with interpulse delay τ_1 varied by $\Delta\tau_1$ = 16 ns. An 8-step phase cycling scheme {(x) [x] x_p x} was performed to remove other echoes capable of interfering with the detected signal as suggested by Tait and Stoll[27]. Rectangular pulses with typical lengths of ca. $t_{\pi/2}$ = 15 ns and t_π = 30 ns were used as observer pulses. The pump pulse was a 90 MHz broad HS{1,1} pulse with t_π = 100 ns and β/t = 8 that was set to match the nitroxide and resonator maximum at a frequency offset of +90 MHz to the observer pulses. It was calculated with MATLAB (version 2021b) using the function "pulse" of the easyspin software package[28]. Depending on the signal-to-noise of each sample, 2 - 15 scans were recorded with 20 shots per point over 1 – 10 h (Table S5).

2.8.2.3 Distance analysis

Individual scans of the DEER measurements were phase-corrected and summed using MATLAB (version 2021b). Shown distance distributions were evaluated with the integrated deep neural network DEERNet (Generic).[29] Uncertainties in the 4p-DEER data analysis with DEERNet are obtained using an ensemble of independently trained neural networks and are shown as 95 % confidence intervals (shaded areas) in the distance distributions (P(r) in Figure 4, Figure S14 - Figure S10)[29,30].

The distance constraints resulting from DEERNet analysis were compared with evaluation by Tikhonov regularization[31,32] with DeerAnalysis[33] (version 2019), which was the golden standard of model-free DEER distance evaluation before one-step approaches that fit the background and distance in a single step were introduced. For the analysis with Tikhonov, the zero time and the starting times for 3-dimensional background fit were determined automatically. The α parameter was determined using the L-curve criterion[33]. All resulting distance distributions were similar to the results from analysis with DEERNet. Some examples of a comparison from DEERNet and Tikhonov L-curve analysis are shown in Figure S13. The resulting distance distributions in Figure S13 were validated using the validation tool included in the DeerAnalysis2019 software to assess the certainty of the distance evaluation. For this validation, new distance distributions were calculated on the same trace by adding up 1.5-fold noise (10 different values), varying the background start between 1 – 3.5 µs (12 values) and the background dimension between 2.0 and 3.5 (4 values). Out of these 480 analyses, fits that exceeded 1.15 rmsd away of the best fit were excluded by using a prune level of 1.15. The remaining distance distributions gives an idea about the accuracy of the analysis method and are depicted as gray shaded areas in Figure S13.

In addition, the newest and 3rd method for DEER distance analysis was used for comparison in Figure S13: DeerLab[34]. DeerLab provides in addition two methods for statistic validation of the resulting distance distributions: uncertainty estimation by covariance matrix and bootstrapping.[34] Figure S13

shows the result from DEER lab in red, using 4-pulse DEER as experiment (ex_4pdeer), a 3D homogenous background function (bg_hom3d), a cutoff at 5 µs of the DEER trace and generalized cross-validation (gcv) criterion for determination[35] of the regularization parameter. The 95 % confidence intervals depicted as red shaded areas.

3 SUPPLEMENTARY DATA

3.1 ITC DATA

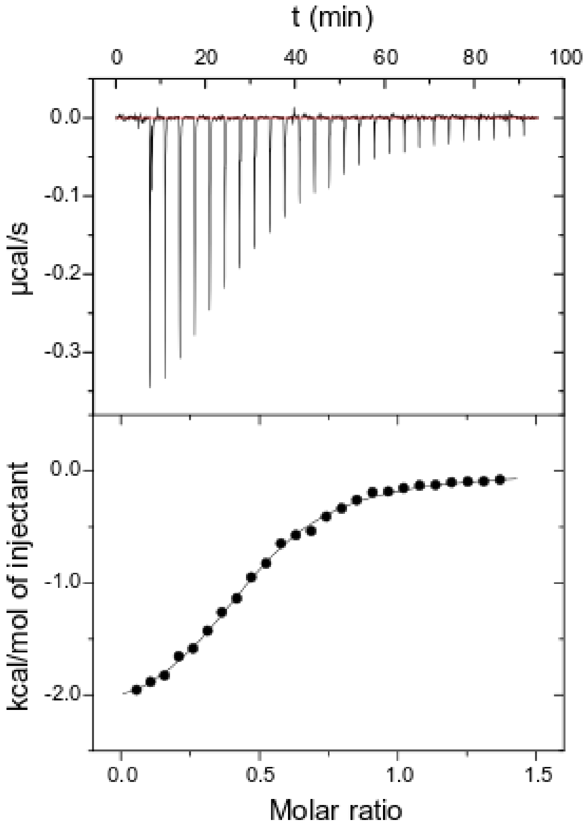

	AdK + AP4A
N	0.47 ± 0.01
K_d (µM)	12.4 ± 0.6
ΔH^0 (kJ mol^{-1})	-10.9 ± 1.8
$-T\Delta S^0$ (kcal mol^{-1})	-17.1 ± 1.9
ΔG^0 (kcal mol^{-1})	-18.0 ± 3.7

Figure S1. ITC thermograms for AP4A binding to *E coli* AdK.
(**Left**) Heat traces were obtained by titrating 1.15 mM AP4A to 168 µM eAdK (wild-type) in 26 steps. (**Right**) the summarized thermodynamic parameters for the interaction of AdK with AP4A (n = 4, mean ± standard deviation) after a single-site-binding isotherm was fitted. The ITC data indicated a K_d = 12.4 ± 0.6 µM of AdK to AP4A. This is in accordance with results from Reinstein *et al.,* indicating a K_d of 43 µM for wild type *E. coli* AdK for AP4A in the presence of 5 mM $MgCl_2$, and a higher affinity of 13 µM for AP4A in the presence of 2.5 mM EDTA (without Mg^{2+}) using displacement experiments with a fluorescent AP5A analogue.[36] Note that we did not attempted to measure the K_d values in the presence of Mg^{2+}.

3.2 CRYSTALLOGRAPHY DATA

Here, we provide a more detailed reasoning why we think that 2 ADP molecules resemble more closely to the electron density of the ligand when *E. coli* AdK is co-crystallized with AP4A (Figure S2).

The electron densities for the ligands in the active centers of both monomers in the asymmetric unit are well-defined and thus allow a distinction between AP4A and ADP (Figure S2A, C). In more detail:

i) No electron density connecting the two β-phosphorus atoms (via an oxygen atom) are observed - which would be expected to be noticeable in this well-defined density and in case of AP4A binding. Instead, two clearly separated electron densities are observed, which suggests two distinct molecules.

ii) The electron density for four (oxygen) atoms can be observed around the density of each β-phosphorus atom, which is expected for the binding of two ADP molecules, whereas binding of AP4A would result in electron density for seven (oxygen) atoms around both β-phosphorus atoms. This is supported by modelling trials of ADP as well as AP4A. Placement of two ADP molecules (Figure S2A) explains the observed electron densities considerably better than the placement of an AP4A molecule (Figure S2C).

Furthermore, the modelling attempts show that the placement of AP4A would result in an unusual geometry around the two β-phosphates, while building of two ADPs shows expected geometries in these molecules (Figure S2B, D):

iii) The O-βP-O angle in the modelled AP4A (Figure S2D) is for example larger than the comparable angles for ADP (130° compared to 107°). This bond angle seems not impossible when comparing to *ab initio* calculations on methyl di- and triphosphates that report O-P-O angles of 92° - 136°.[37] However, comparing to similar angles in previously determined protein structures with AP4A, the angles are in the 102° - 114° range (PDB ID: 2E20 and 2C9Y)[38]. In the crystal structure of *E. coli* AdK co-crystallized with AP5A, the OPO bond angles of the β- or β'- phosphates were between 105° and 116° (PDB ID: 1AKE)[39].

iv) Another example in the modelled AP4A are the bond lengths of the β-, β'- phosphorus atoms and the oxygen atom bridging them, which are 1.72 and 1.73 Å. Here, *ab initio* calculations report bond lengths of 1.61 - 1.72 Å[37] and in previously determined structures the P-O-P bonds are between 1.62 - 1.65 Å long (PDB ID: 2E20 and 2C9Y)[38]. In other *E. coli* AdK structures co-crystallized with ADP, P-O-P bond lengths in the range of 1.63 - 1.66 Å were reported[40].

Consequently, building of two ADPs results in molecule geometries that are more in line with the expected values, than the strained geometries in the modelled AP4A molecule, which has both unusual bond angles and bond lengths. Therefore, we conclude that 2 ADP molecules rather than AP4A are bound to AdK in the crystal.

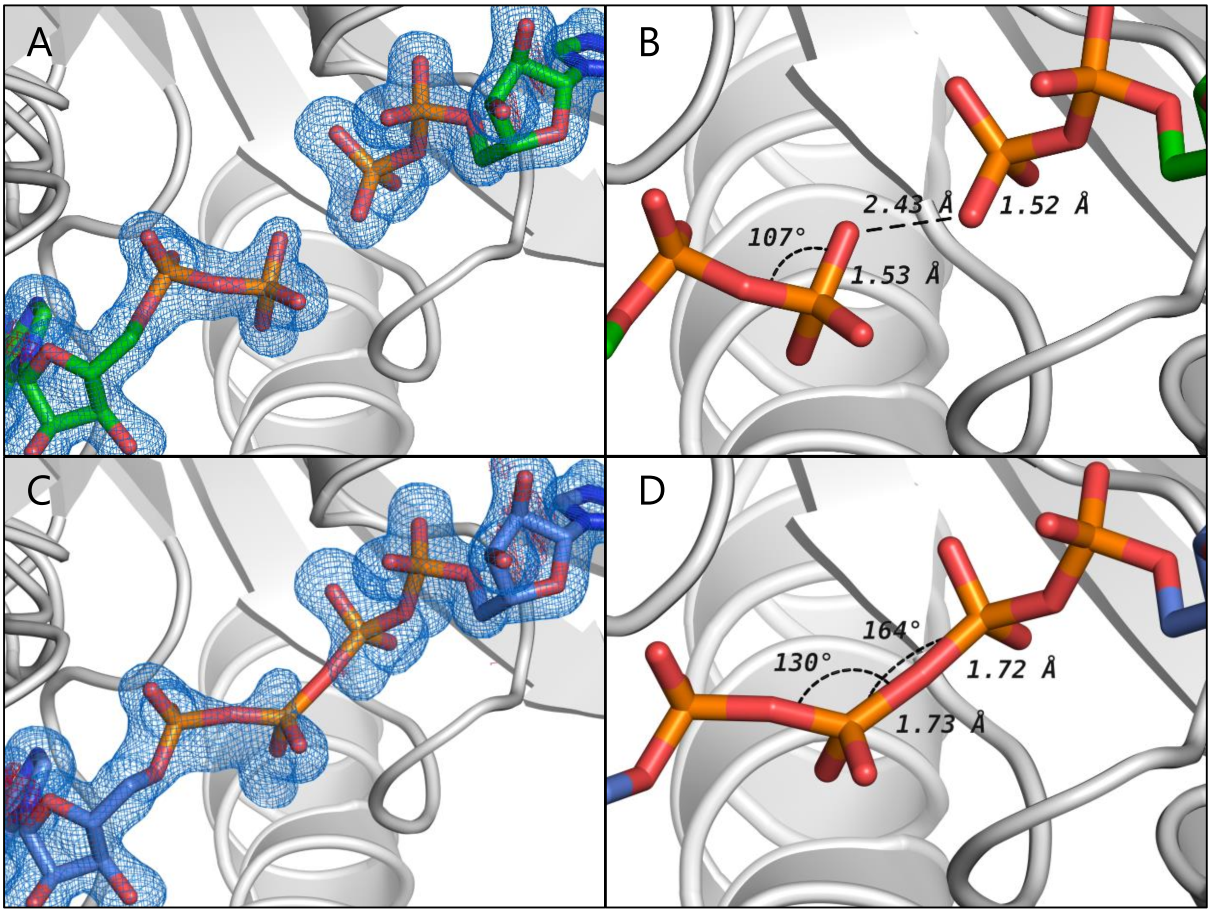

Figure S2. Modelling of ADP and AP4A into the X-ray structure of *E. coli* AdK co-crystallized with AP4A (PDB ID: 8CRG). The Fo-Fc difference electron density maps from a simulated annealing refinement with omitted ligands are shown in blue and are contoured at 3σ. (**A**) Two ADP molecules modelled into the electron densities of the ligands. (**B**) Zoom to the β-phosphate group of both ADPs with exemplary O-P-O angle and distances. (**C**) AP4A modelled into the same electron densities. (**D**) Stretched angles and long P-O-P distances for the β- and β'-phosphate group of the modelled AP4A are indicated in the zoom. (A, B) are shown in the main text as Fig. 2A, B

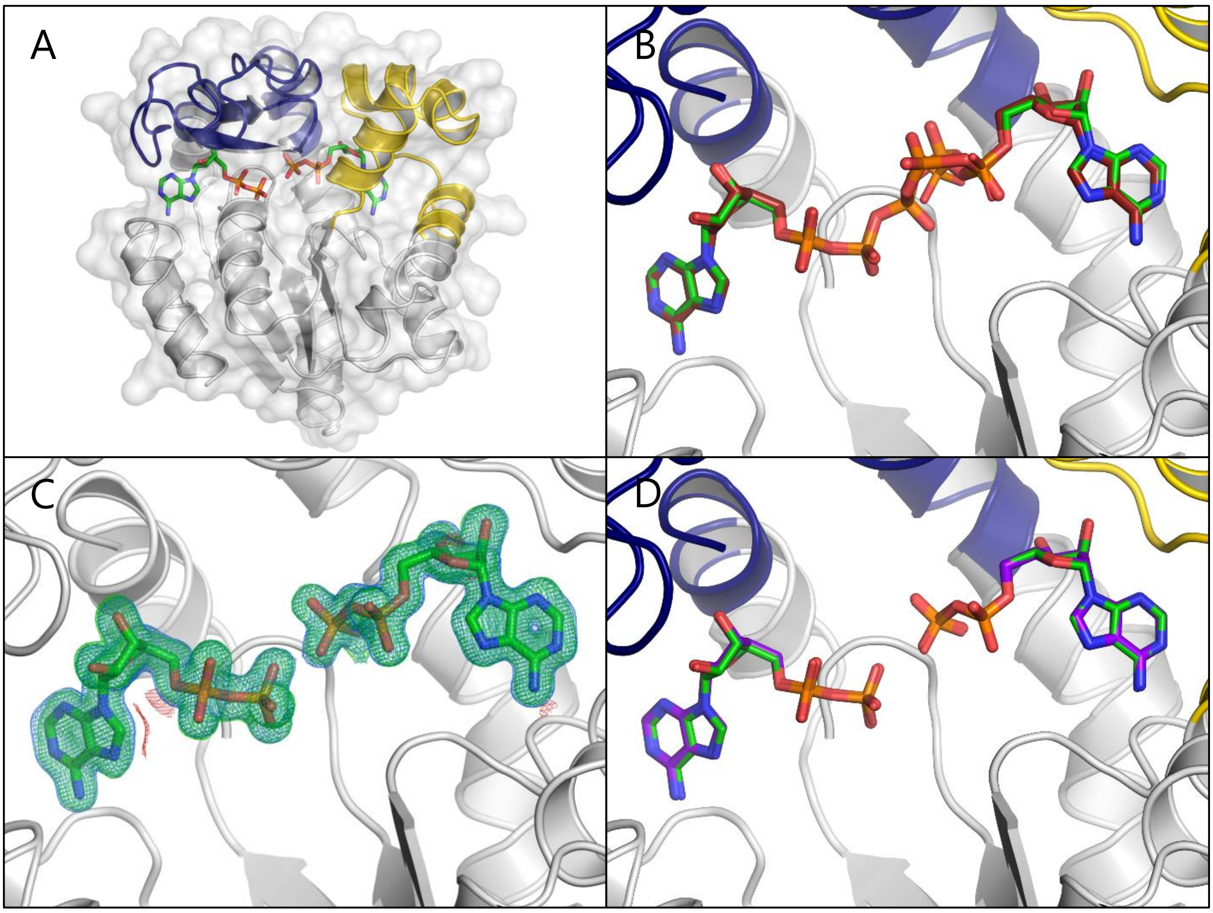

Figure S3. Crystal structure of *E. coli* AdK co-crystallized with AP4A.
(**A**) Overview of the protein structure with the two ADP molecules modelled into the active center. The ATPlid (blue), AMPbd (yellow) and the structure of 2 ADP molecules (green, stick representation) are highlighted. PDB ID: 8CRG. (**B**) Comparison of the two ADPs (green) with AP5A (red) of an AdK-AP5A complex structure (PDB: 1AKE[39]). (**C**) Electron density maps of the two ADP ligands. The 2Fo-Fc map of the final refinement (blue) is contoured at 1 σ. The Fo-Fc difference map from a simulated annealing refinement with omitted ligands (green and red) is contoured at 3 σ. (**D**) Comparison of the two ADPs (green) with the ADP molecules (purple) of another AdK-ADP complex structure (PDB: 7APU[40]).

Table S2: Data collection and refinement statistics for the *E. coli* Adk co-crystallized with AP4A. Matthews coefficients and solvent content estimates were calculated with matthews_coef from ccp4[41,42]. Average B-factors were calculated with baverage from ccp4. Values in parentheses are for the highest resolution shell.

Data collection statistics	
Beamline	BioMAX (MAX IV)
Detector	Dectris Eiger 16M
Wavelength (Å)	0.976254
Space group	$P\ 2_1$
Unit cell (Å)	a = 56.81, b = 77.36, c = 59.60, β = 95.48°
Matthews coefficient ($Å^3Da^{-1}$)	2.77
Solvent content (%)	55.6
Molecules/ASU	2
Resolution range (Å)	37.61 – 1.49 (1.53 – 1.49)
No. of reflections	
Measured	285900 (21040)
Unique	83060 (6130)
Redundancy	3.4 (3.4)
Completeness (%)	99.2 (98.9)
R_{meas} (%)	4.3 (108.5)
R_{merge} (%)	3.7 (86.8)
R_{pim} (%)	2.3 (54.2)
$\langle I\rangle/\langle\sigma(I)\rangle$	15.31 (1.53)
CC(1/2) (%)	99.9 (51.5)
Wilson B-factor ($Å^2$)	30.67
Mosaicity (°)	0.063
Refinement statistics	
R_{work} factor (%)	16.32
R_{free} factor (%)	19.07
No. of reflections in test set	2077 (2.5 %)
No. of non-H atoms	
Protein (chain A/B)	1763/1764
ADP	108
other ligands	26
water	502
Average B-factor ($Å^2$)	
Protein (chain A/B)	31.29/32.31
ADP	22.17
other ligands	83.61
water	38.95
RMSD bond length (Å)	0.009
RMSD bond angles (°)	1.297
Ramachandran regions (%)	
favored regions	100.0
allowed regions	0.0
outliers	0.0
Rotamer outliers [%]	0.0
Clashscore	1.63

3.3 ^{31}P NMR DATA

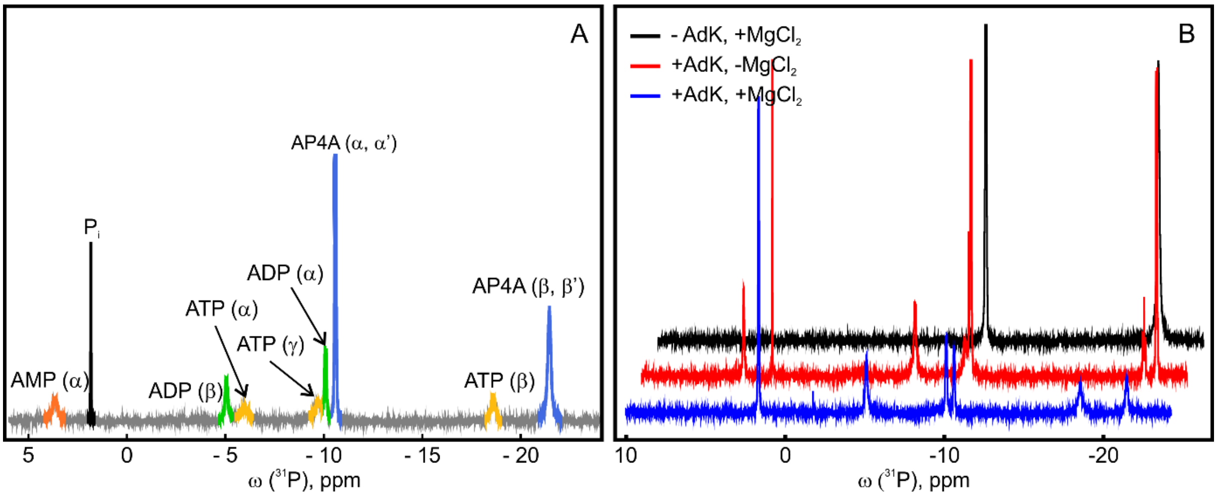

Figure S4. Peak assignment of ^{31}P NMR spectra for the AP4A breakdown by *E. coli* AdK.
(**A**) ^{31}P NMR spectra of 200 µM wild-type AdK with 1 mM AP4A, recorded after 78 h reaction time at 295 K. The different breakdown products are indicated. (**B**) AP4A breakdown in the presence and absence of Mg^{2+}. ^{31}P NMR spectra of 1 mM AP4A with either only $MgCl_2$ (-AdK, +$MgCl_2$, black, after 160 h), only 200 µM AdK (+AdK, -$MgCl_2$, red, after 160 h), or 200 µM AdK and 2 mM $MgCl_2$ (+AdK, +$MgCl_2$, blue, after 78 h). No breakdown is observed for the sample without AdK. The samples containing AdK with or without $MgCl_2$ shows different degrees of breakdown.

3.4 CD DATA

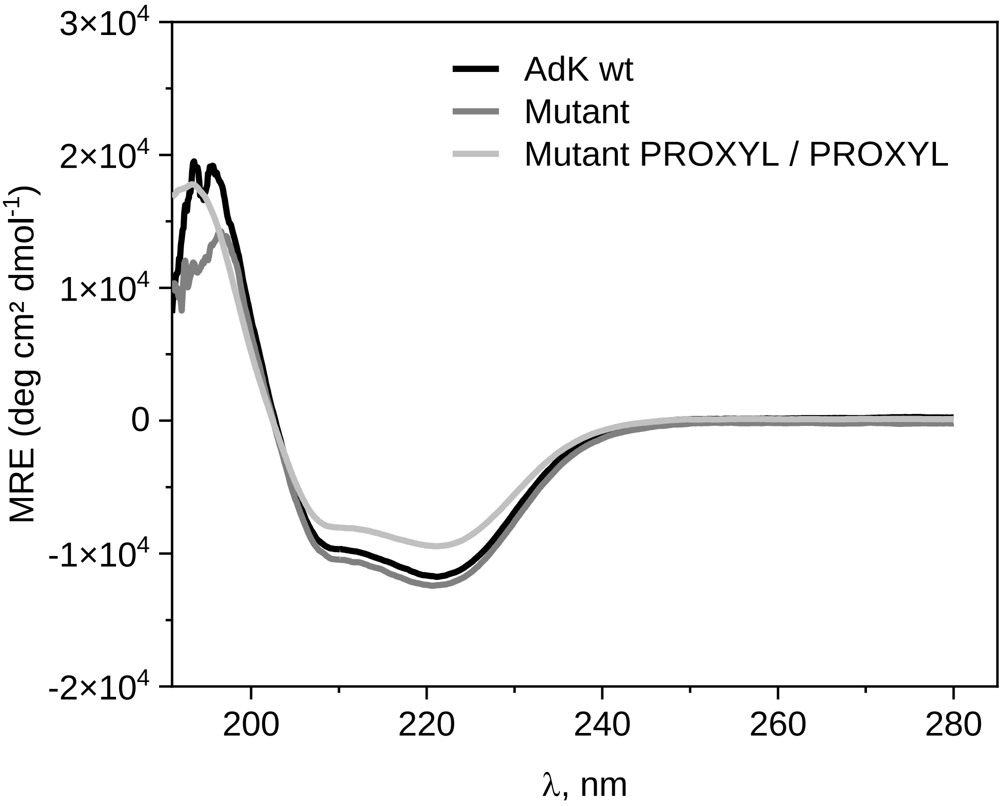

Figure S5: CD spectra of unlabeled and labeled *E. coli* AdK.
Circular dichroism (CD) spectra of *E. coli* AdK wild-type (wt, black) and the unlabeled double mutant eAdK_Lys50pPrF_Val148Cys (mutant, dark gray) are compared to the mutant doubly labeled at position Lys50pPrF with azido-PROXYL and at position Val148Cys maleimido-PROXYL (mutant_ PROXYL / PROXYL, light gray). Spectra are normalized to the mean residue ellipticity (MRE). No qualitative change to the spectral shape is observed in the CD-spectra between wild-type, mutant and labeled mutant, indicating that both mutation and labeling does not interfere with the secondary structure of the protein. Small quantitative deviations are attributed to inaccuracies in the photometric protein concentration determination.

3.5 ENZYMATIC ASSAY & IN-SILICO LABELLING

Table S3. Enzymatic activity of doubly spin-labeled AdK.
Results from coupled enzymatic assay of AdK_Lys50pPrF/Val148Cys doubly labeled with PROXYL with a Michaelis-Menten fit for ATP concentrations in the range of 0 to 1.5 mM ATP. Errors give the standard deviations from 3 replicates. As comparison of the kinetic parameters for AdK doubly labeled with the nitroxide spin labels (AdK_2x NO + ATP) and wild type AdK (AdK_wt + ATP) data for the catalytic efficiency k_{cat}, the Michaelis-Menten constant K_m and the specificity factor k_{cat}/K_m are given. *Values from reference[43].

	AdK_2x NO + ATP	AdK_wt + ATP*
k_{cat}, s^{-1}	52 ± 4	360 ± 10
K_m, µM	76 ± 24	63 ± 7
K_{cat}/K_m, s^{-1} μM^{-1}	0.7 ± 0.2	5.7 ± 0.7

Compared to wild type AdK, the catalytic turn-over is reduced by factor 7 when the enzyme is doubly labeled with azido-PROXYL at position Lys50pPrF in the AMPbd and with maleimido-PROXYL at position Val148Cys (AdK_2x NO + ATP, section 2.7). The reduction in maximal catalytic rate is either because the label at K50 interferes with substrate binding or because the spin labels, even when sterically small, might slow down the motions required for efficient catalysis at the ATPlid and AMPbd.

From in-silico labelling[44] (i.e., attaching label rotamers to the crystal structures of open and closed AdK), we do not expect large steric hindrance (Figure S6). This is supported by the K_M value that increased by (only) a factor of 1.2 for the labeled mutant compared to the wild-type (increase in K_M indicates a decreased binding strength). Thus, the binding affinity for the substrates ATP and AMP seems to be nearly unaffected by the double spin labeling.

Also, since only the protein conformation (and not the catalytic activity) was investigated using the doubly labeled AdK with excess of ligands, the found differences were not deemed important.

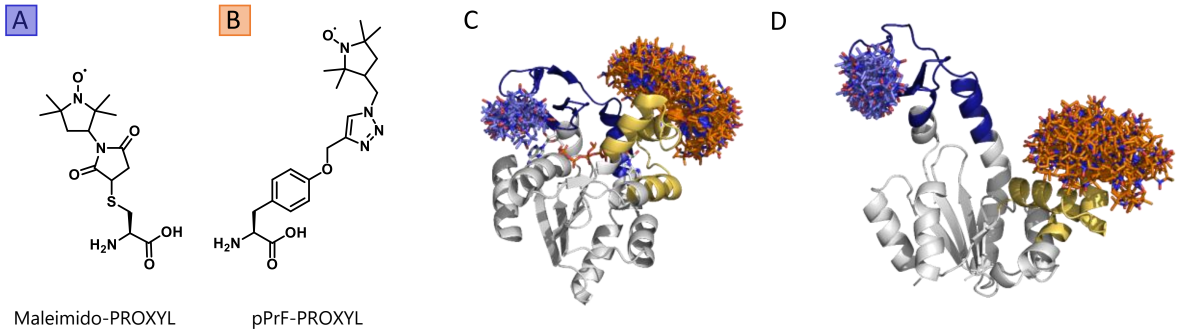

Figure S6. ***In-silico*** **labelling of** ***E. coli*** **AdK.**
Structures of (**A**) Maleimido-PROXYL and (**B**) pPrF-PROXYL used for rotamer generation with MtsslWizard[44]. Tight Van-der-Waals (VdW) restraints were chosen for labelling at V148, loose VdW restraints for labelling at K50, resulting in 30-131 rotamers. (**C**) Depiction of the simulated rotamer structures on AP5A-bound enzyme (PDB ID: 1AKE[39]) and (**D**) the apo enzyme (PDB ID: 4AKE[26]). The pPrF-PROXYL at K50 are marked as orange sticks and Maleimido-PROXYL at V148 in blue. The ATPlid is colored in dark blue, the AMBbd in yellow. No obvious interference of the labels with the substrate binding domain is observed.

3.6 EPR

3.6.1 Labelling controls

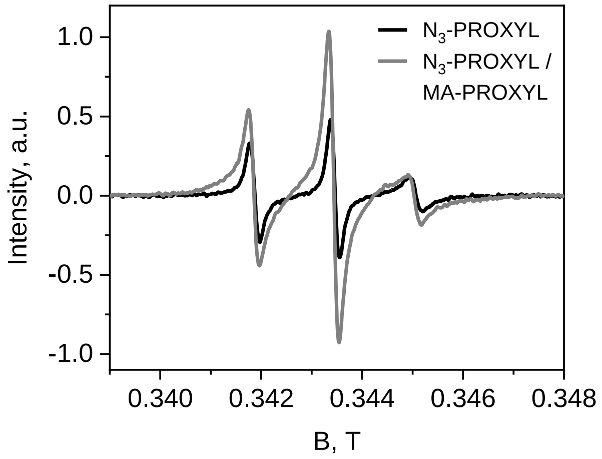

Figure S7. Cw EPR spectra of singly and doubly labeled AdK.
Cw spectra of AdK labeled with azido-PROXYL (black) and subsequently with maleimido-PROXYL (gray). 16 scans were recorded and smoothed with a 10 point Savitzky-Golay filter using Origin (version 2022). Labeling degrees of 50 -70 % for CuAAC labeling at position Lys50pPrF, 80 - 100 % for maleimido-PROXYL at position Val148Cys were obtained.

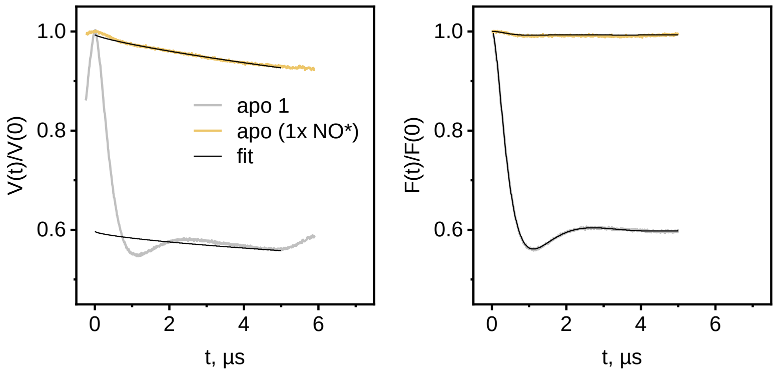

Figure S8: Control for unspecific labeling: 4p-DEER measurement from singly and doubly labeled *E. coli* AdK.
4p-DEER raw data normalized to the maximum (V(t)/V(0), left), after background correction (F(t)/F(0), middle) and resulting distance distribution normalized to the maximum (P(r)/P(max), right) for 20 µM of doubly PROXYL labeled AdK (apo 1, gray) and singly maleimido-PROXYL labeled AdK (apo (1x NO*, beige). Details are listed in Table S5. All DEER curves were analyzed with DEERNet[29] within the DeerAnalysis software[33] (version 2019). A low modulation depth of 0.7 % was found for the control labeled only with maleimido-PROXYL (1x NO*, beige), confirming a very low degree of unspecific labeling of *E. coli* AdK at C77 under given conditions.

3.6.2 Quantification of the lid-to-lid EPR distance measurements

Single Gaussians were fitted to the distance distributions representing the open and closed AdK conformation. We used the AP5A bound AdK as reference for closed enzyme. For the open conformation, we observed a shoulder at smaller distances (3 nm) for the enzyme without ligand that was pronounced to different extend between the samples (compare apo 1 and apo 3, Figure S9). We therefore assumed that part of the enzyme is closed even in absence of any ligand, as reported earlier[45]. Therefore, we used a Gaussian that takes only the long distances into account (dashed red line, Figure S9).

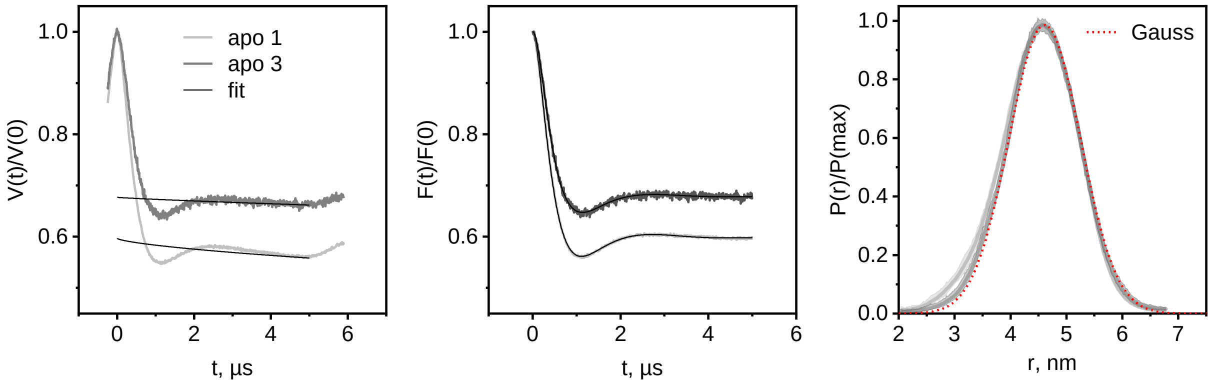

Figure S9. 4p-DEER data of apo *E. coli* AdK.
4p-DEER raw data normalized to the maximum (V(t)/V(0), left), after background correction (F(t)/F(0), middle) and resulting distance distribution normalized to the maximum (P(r)/P(max), right) are shown for two samples with 20 µM AdK without ligand (apo 1 and apo 3). Details are listed in Table S5. All DEER curves were analyzed with DEERNet[29] within the DeerAnalysis software[33] (version 2019). Interestingly, the shoulder at approximately 3 nm in the distance distribution P(r) of apo 1 is less pronounced in the sample apo 3. A single Gaussian was fitted to the main peak (red dotted lines) for quantification of the open conformation.

We used a Gaussian analysis of a two-state model to describe the distance distribution for the AP4A-bound AdK as superposition of the closed and open state. To this end, the mean value and standard deviation of the 2 single Gaussians were kept fixed (Table S4) and only the amplitudes were varied. The resulting populations of open and closed states are listed in Figure S10 A.

Table S4. Parameter for Gauss fitting.
r_{mean}: mean value and σ standard deviation of both Gaussians representing the closed and open AdK enzymes.

	Gauss 1 (closed AdK)	***Gauss 2 (open AdK)***
r_{mean} *(nm)*	3.62	4.61
σ *(nm)*	0.56	0.64

A

Ligand	*% closed*	*% open*
apo	11 ± 1	89 ± 1
AP4A	42 ± 2	58 ± 2
AP5A	97 ± 5	3 ± 5

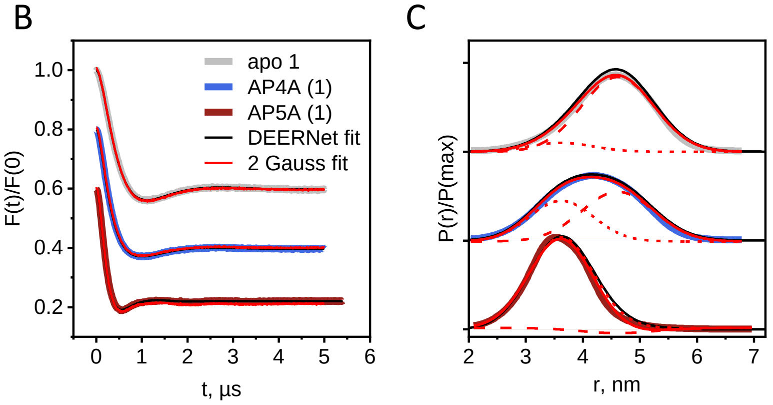

Figure S10. Quantification of open versus closed conformation of AP4A bound AdK for 4p-DEER data.
(**A**) Percentages of open versus close *E. coli* AdK conformation that results from Gaussian fitting of distance constraints obtained from 4p-DEER EPR distance measurements. The mean ± the difference between two independent measurements (see Figure S14, Table S5 are shown. (**B**) Fits to the background corrected DEER data of 20 µM AdK (apo, gray), AdK with 2 mM of AP4A (blue) and AdK with 100 µM AP5A (red) are compared. Fits from DEERNet fitting procedure (the method chosen for all 4p-DEER data in this manuscript) are shown in black and fitting with 2 Gaussian in red. Both fits are very similar and represent the data well. (**C**) Depiction of the 2 single Gaussians scaled to percentage used for fitting (dashed and dotted red lines) and their sum (red line) over the distance distribution P(r) obtained from DEERNet fitting. Small deviations between both fitting procedures are mainly found for AdK with AP5A, where the Gauss fit representing fully closed enzyme is deviating at 4.5 nm from the DEERNet result.

3.6.3 AP4A binding mode

Because AP4A is a symmetric molecule, binding to both binding sites (i.e., to AMPbd or ATPlid separately or simultaneously) might be possible.

To test whether AP4A only binds to one of the two binding sites, we compared the EPR distance distribution obtained for the AP4A-bound enzyme with (i) AdK bound only to AMP and (ii) AdK bound only to ATP (Figure S11 and Figure S12).

Hereby, we assumed that the AMP and ATP bind to their respective binding domains (i.e., the AMPbd and the ATPlid) and not to both binding domains. This was shown before by NMR, and AMP or ATP binding lead to an equilibrium of open and closed conformations of the respective binding domains, while the other binding domain remains in open conformation[46].

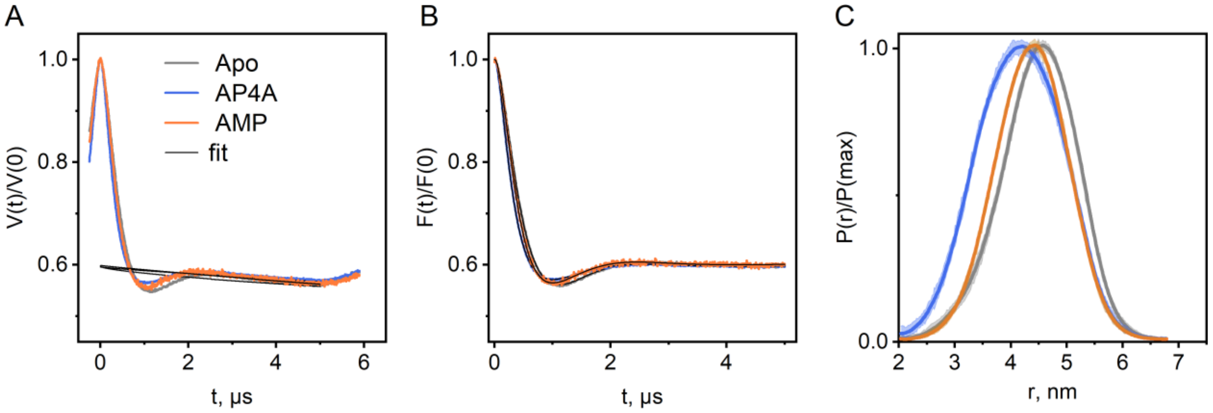

Figure S11. Comparison of EPR DEER data of apo AdK and bound to AMP or AP4A.
(A) 4p-DEER raw data normalized to the maximum (V(t)/V(0)), (B) after background correction (F(t)/F(0)) and (C) resulting distance distribution normalized to the maximum (P(r)/P(max)), for 20 µM of doubly PROXYL labeled AdK (apo, gray), with

2 mM AP4A (blue) or with 20 mM AMP (orange). Details are listed in Table S5. All DEER curves were analyzed with DEERNet[29] within the DeerAnalysis software[33] (version 2019). The distance distributions for AMP-bound AdK shows a larger population of open enzymes than when AP4A-bound.

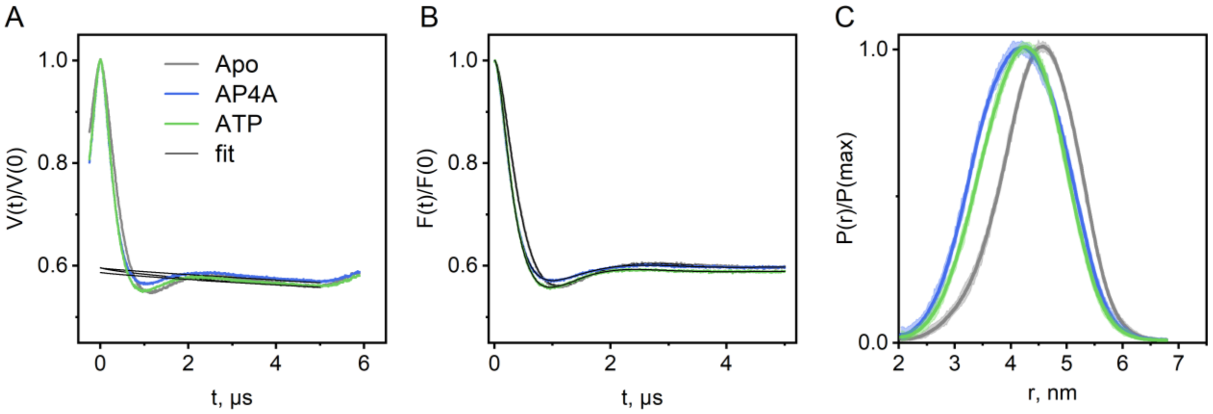

Figure S12. Comparison of EPR DEER data of apo AdK and bound to ATP or AP4A.
(A) 4p-DEER raw data normalized to the maximum (V(t)/V(0)), (B) after background correction (F(t)/F(0)) and (C) resulting distance distribution normalized to the maximum (P(r)/P(max)), for 20 µM of doubly PROXYL labeled AdK (apo, gray), with 2 mM AP4A (blue) or with 4 mM ATP (green). Details are listed in Table S5. All DEER curves were analyzed with DEERNet[29] within the DeerAnalysis software[33] (version 2019). The distance distributions for ATP-bound AdK ressembles that of AP4A-bound AdK.

From the Figure S11 and Figure S12 above, discrimination between the conformations of apo AdK, AMP-, ATP- and AP4A-bound AdK is possible.

(i) It becomes clear that closing of the AMPbd alone does not lead to a conformation that is comparable with the AP4A-bound state (Figure S11), as the distance distribution is narrower and shifted towards longer distances for AMP-bound AdK compared to AP4A-bound AdK. Thus, AP4A binding to only the AMPbd is not what we observe. Therefore, we conclude that binding of AP4A occurs at the ATP binding site.

(ii) Figure S12 shows that AP4A-binding lead to a similar distance distribution than ATP-binding but shifted slightly (< 0.1 nm, Table S4) towards smaller distances, indicating more closed enzymes in the presence of AP4A.

From the EPR data, we cannot tell whether this is due to a higher population of enzymes with closed ATPlid or because both substrate binding domains closed simultaneously in some enzymes, while other remained fully open. However, the NMR data shown in Figure 4F in the main text indicate chemical shift changes in both the ATP and AMP binding domains; thus, closing of only the ATPlid can be excluded.

Summarized, we think that both the AMPbd and ATPlid close when binding AP4A, which than leads to hydrolysis.

3.6.4 Controls for reproducibility

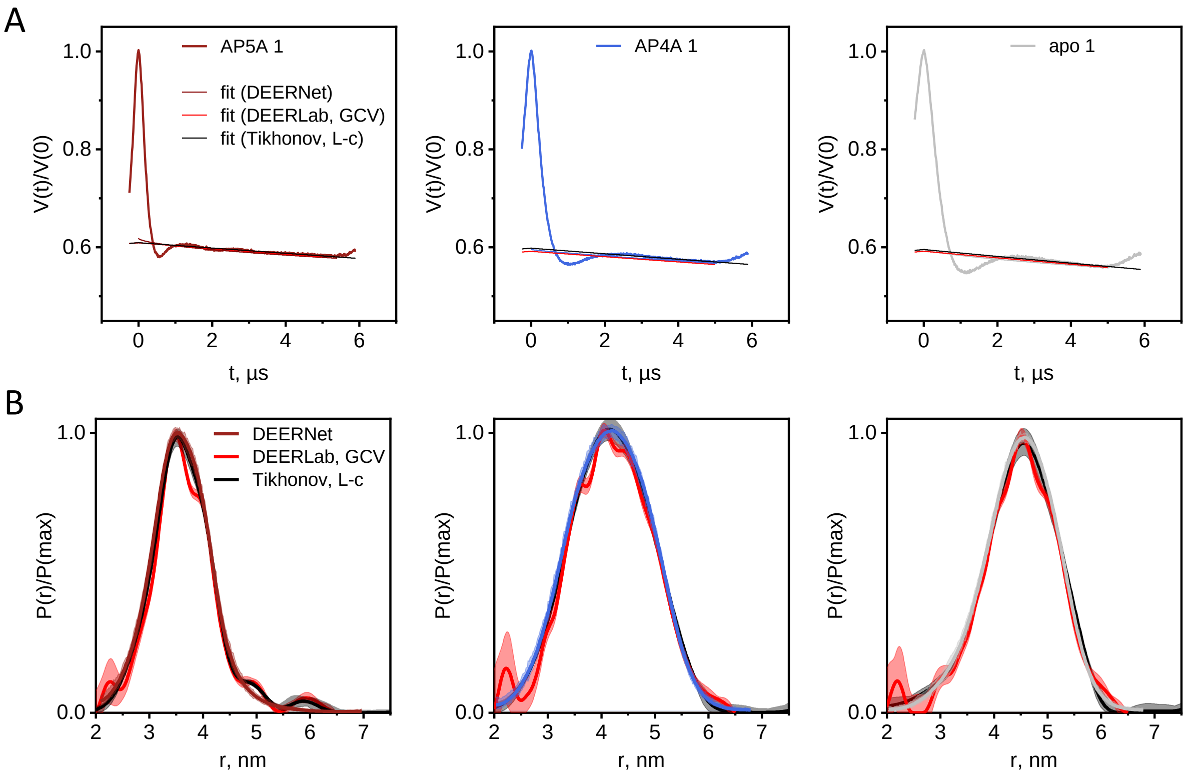

Figure S13. Comparison different analysis methods for EPR distance data.
(**A**) 4p-DEER raw data normalized to the maximum (V(t)/V(0), top) and (**B**) resulting distance distribution normalized to the maximum (P(r)/P(max)) for 20 µM *E. coli* AdK with the different ligands: 100 µM AP5A (left, brown), 2 mM AP4A (blue, middle) and apo (gray, right). Details are listed in Table S5. Analysis was performed with different methods: DEERNet[29] (in respective colors as shown in figures before), with the conventional Tikhonov approach using the automated L-curve criterion[33] (L-c, black) and the newest approach using DeerLab[34], here with the GCV criterion (GCV, red). Details and parameters for the different analysis methods are given in the text above. Similar background fits and resulting distance distributions (Figure S13) imply that the resulting distance distributions are independent on the analysis approach. For the following DEER data evaluations, we used the DEERNet method.

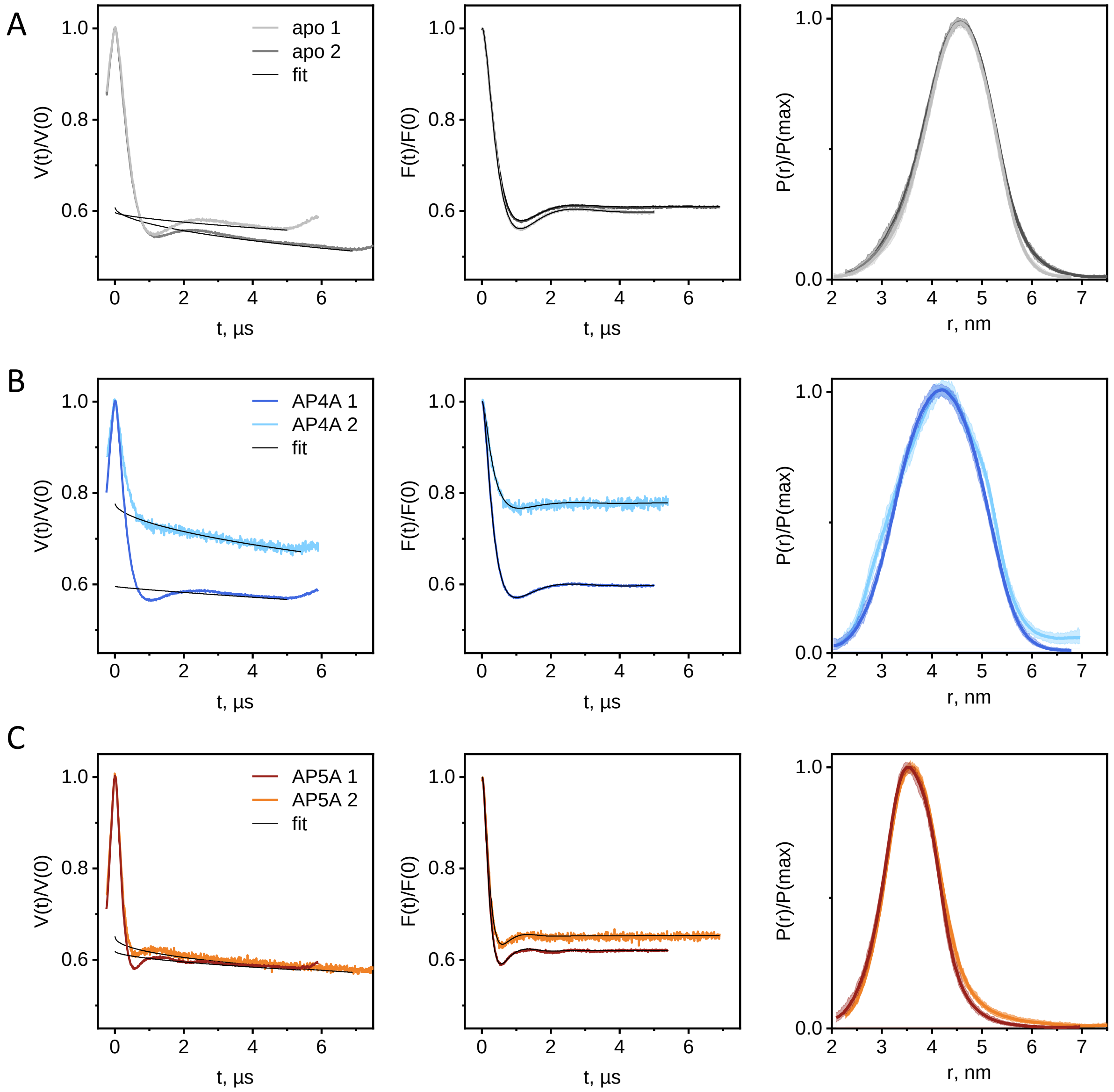

Figure S14. Reproducibility of EPR 4p-DEER data on *E. coli* AdK with different ligands.
4p-DEER raw data normalized to the maximum (V(t)/V(0), left), after background correction (F(t)/F(0), middle) and resulting distance distribution normalized to the maximum (P(r)/P(max), right) are shown for the different ligands (**A**) apo, (**B**) AP4A, (**C**) AP5A. Details and concentrations are listed in Table S5. All DEER curves were analyzed with DEERNet[29] within the DeerAnalysis software[33] (version 2019). The distance distributions P(r) were reproducible for different labeling batches with different double labeling degrees that consequently led to different modulation depths for the form factor F(t)/F(0). Note that different number of scans were accumulated, see Table S5 for details.

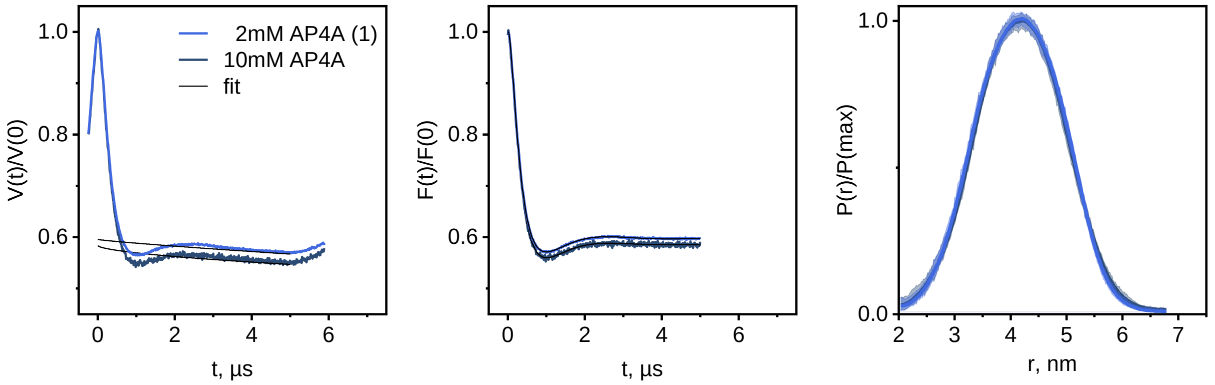

Figure S15. High Excess AP4A.

4p-DEER raw data normalized to the maximum (V(t)/V(0), left), after background correction (F(t)/F(0), middle) and resulting distance distribution normalized to the maximum (P(r)/P(max), right) for 20 µM of doubly PROXYL labeled AdK with 2 mM and 10 mM AP4A (light and dark blue, respectively). Details are listed in Table S5. All DEER curves were analyzed with DEERNet[29] within the DeerAnalysis software[33] (version 2019). The similar distance distributions imply that higher excess of AP4A does not change the enzyme conformation any further and that saturation is reached already with 2 mM AP4A.

Table S5. Parameters of 4p-DEER distance measurements.

<r>: mean distance, s(r): width of distance distribution, λ: modulation depth. Bold samples are measurements shown in the main article and re-used several times in the SI for comparisons.

Ligand	*Sample name*	*c_{AdK} (µM)*	*c_{ligand} (µM)*	*<r> (nm)*	*s(r) (nm)*	*λ (%)*	*Scan no.*	*Analysis method*
-	**apo 1**	**20**	**-**	**4.49**	**0.70**	**40.3**	**5**	**DEERNet**
				4.53	0.92	40.4	5	Tikhonov. L-curve
						40.7	5	DeerLab. GCV
	apo 2	50	-	4.52	0.76	39.2	5	DEERNet
	apo 3	20	-	4.58	0.67	32.3	6	DEERNet
	apo (1x NO*)	20	-	4.70	0.72	0.7	14	DEERNet
AP4A	**AP4A 1**	**20**	**2000**	**4.17**	**0.76**	**40.4**	**5**	**DEERNet**
				4.21	0.87	40.2	5	Tikhonov. L-curve
						40.8	5	DeerLab. GCV
	AP4A 2	45	4000	4.23	0.86	22.3	13	DEERNet
	10 mM AP4A	20	10’000	4.18	0.78	41.6	5	DEERNet
AP5A	**AP5A 1**	**20**	**100**	**3.62**	**0.60**	**38.2**	**15**	**DEERNet**
				3.71	0.78	39.0	15	Tikhonov. L-curve
						39.0	15	DeerLab. GCV
	AP5A 2	50	250	3.75	0.71	34.9	3	DEERNet
AMP	AMP	20	20’000	4.37	0.67	10.1	2	DEERNet
ATP	ATP	20	4’000	4.20	0.72	41.3	15	DEERNet

3.7 ^1H ^{15}N HSQC NMR

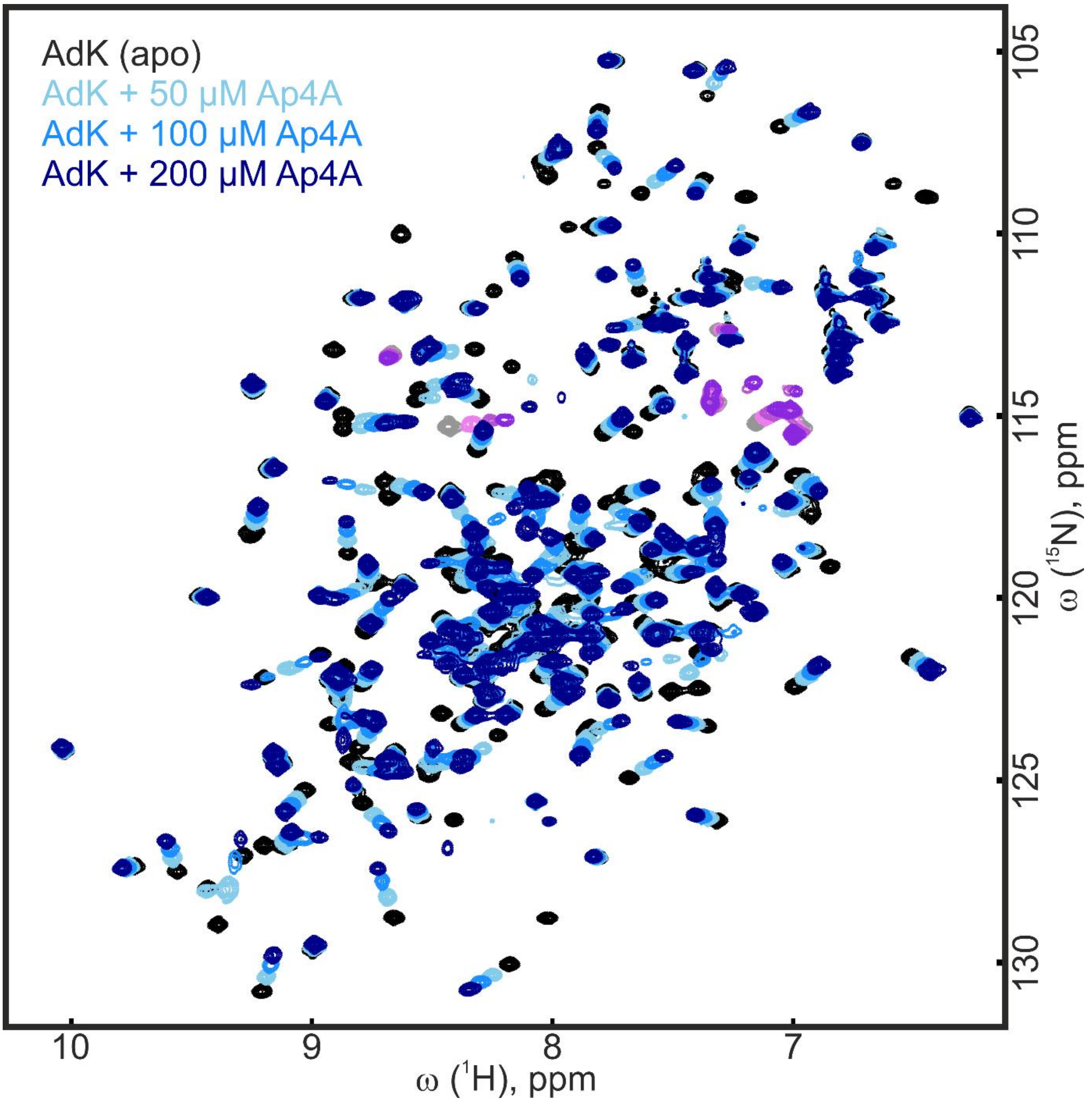

Figure S16: AP4A titration to wt AdK.

Overlay of ^1H ^{15}N HSQC spectra acquired for ^{15}N wt AdK without ligand (apo, black), with 50 µM, 100 µM and 200 µM AP4A (light to dark blue). The gradual resonance shift of some amino acids indicates a gradual conformational change of these residues in dependence on the AP4A concentration.

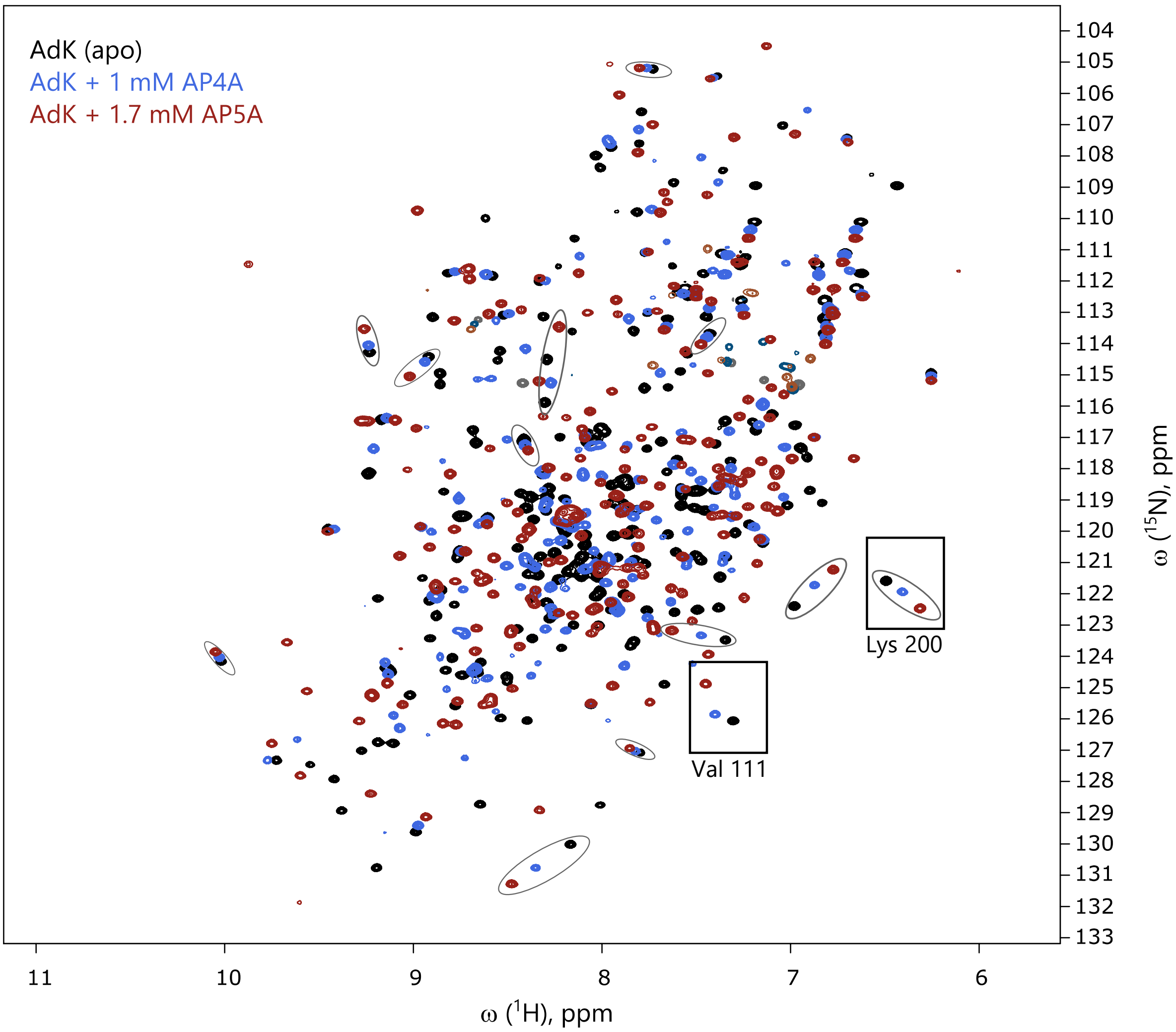

Figure S17. Comparison of ^{1}H ^{15}N HSQC spectra of *E. coli* AdK with AP4A and AP5A.
Overlay of ^{1}H ^{15}N HSQC spectra acquired for wild-type ^{15}N AdK without ligand (apo, black), with 1 mM of AP4A (blue) and 1.7 mM of the inhibitor AP5A (red). Some amino acid residue peaks with large chemical shift differences are circled as guide to the eye, showing that the chemical shifts differences from apo are highest in the AP5A bound form. Exemplary residues Val111 and Lys200 for Figure S18 are highlighted by boxes. The comparison of these three spectra hints that AdK adopts a conformation that is neither open, apo-like, nor fully closed as it is the case when AP5A is bound. (Same spectra as in the main text, figure 4D).

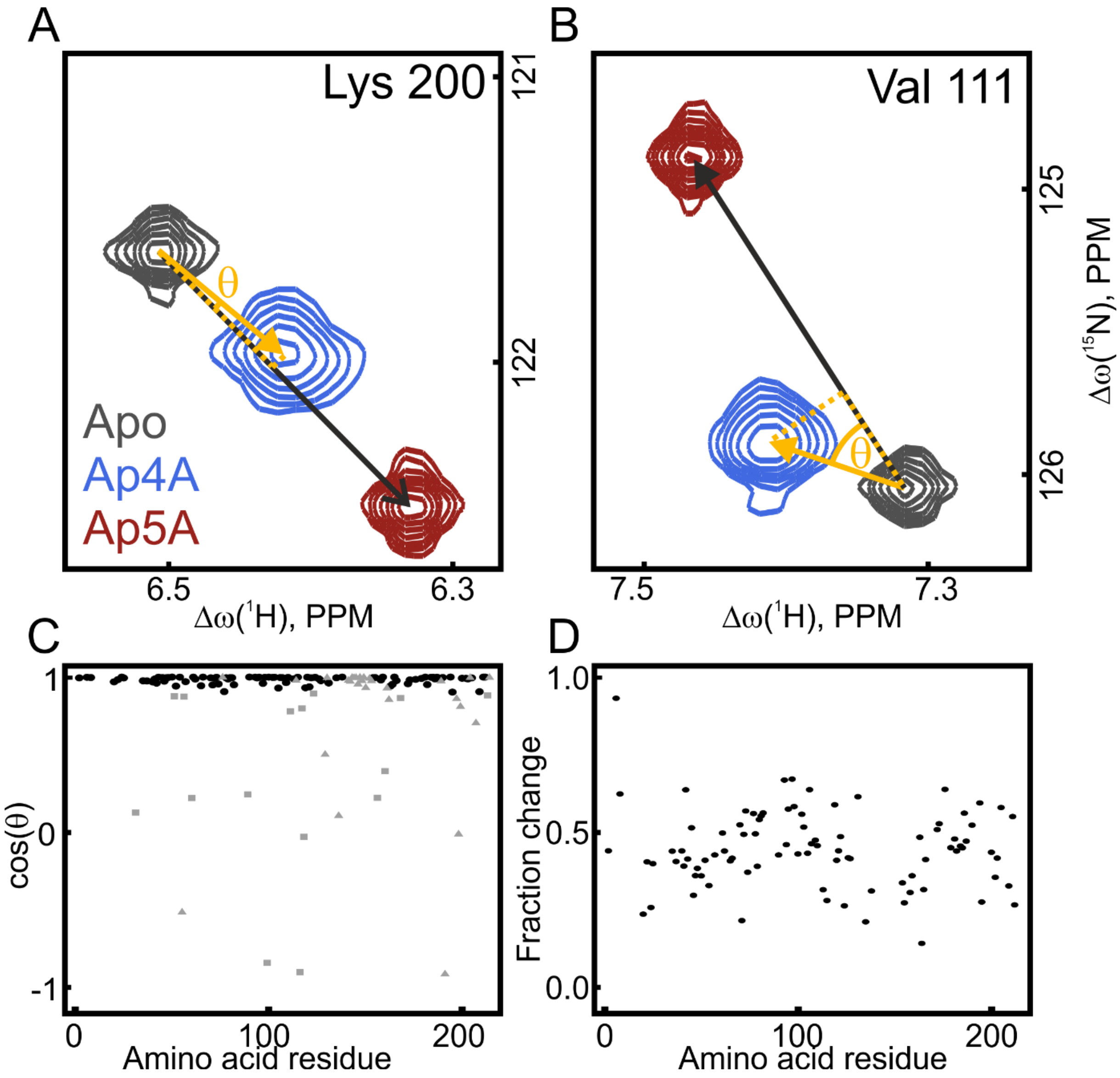

Figure S18: Projection analysis of chemical shifts induced by AP4A and AP5A.

(**A**) An example of an amino acid residue included in the analysis, lysine 200 and (**B**) a residue not included in the analysis, Val111. The angle (θ) between the black reference vector between the apo signal (blue) and the AP5A signal (red), and the yellow activation vector between the apo and the AP4A signals determines how much of the chemical shift induced by AP4A binding can be attributed to the same conformal change as AP5A binding. A cosine of this angle above 0.9 denotes that the angle is small enough to be reliable included in the analysis. In this case for lysine 200, the cos(θ) is 0.99 and for Val111, the cos(θ) is 0.81. The dashed black/yellow part of the reference vector is the projected AP4A vector and the ratio between the length of this and the length of the full vector. (**C**) All cosine of the projection angles θ between AP4A and AP5A projection vectors. The black dots represents projections that were included in the analysis, thus fulfilling the requirements of a cos (θ) higher than 0.9 and a minimum shift difference of 0.05 ppm. Gray dots represent residues excluded from the analysis, with squares marking residues with a cos (θ) lower than 0.9 and triangles marking residues with an absolute value of the chemical shift lower than 0.05 ppm. (**D**) Size of the projected AP4A chemical shift vector on the AP5A chemical shift vector of all residues included in the analysis. The average projected chemical shift induced by AP4A binding is 0.44 ± 0.13 of the chemical shifts induced by AP5A.

4 ABBREVIATIONS

4p-DEER:	4-pulse double electron-electron resonance
AP4A	Diadenosine 5’,5’’’-P^1,P^4-tetraphosphate
AP5A	Diadenosine 5’,5’’’-P^1,P^4-pentaphosphate
Azido-PROXYL:	3-(Azido)-2,2,5,5-tetramethyl-1-pyrrolidinyloxy
BTTAA:	2-(4-((bis((1-(tert-butyl)-1H-1,2,3-triazol-4-yl)methyl)amino)methyl)-1H-1,2,3-triazol-1-yl)acetic acid
CuAAC:	Copper(I)-catalyzed azido-alkyne click reaction
EDFS:	Echo-detected field sweep
HSQC:	Heteronuclear single quantum coherence
iTC:	Isothermal titration calorimetry
Maleimido-PROXYL:	3-Maleimido-2,2,5,5-tetramethyl-1-pyrrolidinyloxy
MOPS:	3-(Morpholin-4-yl)propane-1-sulfonic acid
PBS:	Phosphate-buffered saline
pPrF:	*para*-propargyloxy-*L*-phenylalanine
rmsd:	root-mean square deviation
r.t.:	room temperature
TCEP:	Tris(2-carboxyethyl)phosphine
TRIS:	2-Amino-2-(hydroxymethyl)propane-1,3-diol
wt	wild-type

5 REFERENCES

1. Kucher, S. *et al.* Orthogonal spin labeling using click chemistry for in vitro and in vivo applications. *J. Magn. Reson.* **275**, 38–45 (2017).

2. Reinstein, J., Brune, M. & Wittinghofer, A. Mutations in the Nucleotide Binding Loop of Adenylate Kinase of Escherichia coli. *Biochemistry* **27**, 4712–4720 (1988).

3. Rogne, P. *et al.* Molecular mechanism of ATP versus GTP selectivity of adenylate kinase. *PNAS* **115**, 3012–3017 (2018).

4. Deiters, A. *et al.* Adding Amino Acids with Novel Reactivity to the Genetic Code of Saccharomyces Cerevisiae. *J. Am. Chem. Soc.* **125**, 11782–11783 (2003).

5. Widder, P., Berner, F., Summerer, D. & Drescher, M. Double Nitroxide Labeling by Copper-Catalyzed Azide–Alkyne Cycloadditions with Noncanonical Amino Acids for Electron Paramagnetic Resonance Spectroscopy. *ACS Chem. Biol.* **14**, 839–844 (2019).

6. Liu, C. C. & Schultz, P. G. Adding New Chemistries to the Genetic Code. *Annu. Rev. Biochem.* **79**, 413–444 (2010).

7. Ursby, T. *et al.* BioMAX - the first macromolecular crystallography beamline at MAX IV Laboratory. *J. Synchrotron Radiat.* **27**, 1415–1429 (2020).

8. Kabsch, W. XDS. *Acta Crystallogr. Sect. D Biol. Crystallogr.* **66**, 125–132 (2010).

9. Winn, M. D. *et al.* Overview of the CCP4 suite and current developments. *Acta Crystallogr. Sect. D Biol. Crystallogr.* **67**, 235–242 (2011).

10. Liebschner, D. *et al.* Macromolecular structure determination using X-rays, neutrons and electrons : recent developments in Phenix. *Acta Crystallogr. Sect. D, Struct. Biol.* **75**, 861–877 (2019).

11. Vagin, A. & Teplyakov, A. MOLREP : an Automated Program for Molecular Replacement. *J. Appl. Theor. Phys. Res.* **30**, 1022–1025 (1997).

12. Murshudov, G. N., Vagin, A. A. & Dodson, E. J. Refinement of Macromolecular Structures by the Maximum-Likelihood Method. *Acta Crystallogr. Sect. D* **53**, 240–255 (1997).

13. Afonine, P. V. *et al.* Towards automated crystallographic structure refinement with phenix.refine. *Acta Crystallogr. D. Biol. Crystallogr.* **68**, 352–367 (2012).

14. Emsley, P., Lohkamp, B., Sott, W. G. & Cowtan, K. Features and development of Coot. *Acta Crystallogr. Sect. D Biol. Crystallogr.* **66**, 486–501 (2010).

15. Long, F. *et al.* AceDRG: a stereochemical description generator for ligands. *Acta Crystallogr. Sect. D, Struct. Biol.* **73**, 112–122 (2017).

16. Yuan, S., Chan, H. C. S. & Hu, Z. Using PyMOL as a platform for computational drug design. *Wiley Interdiscip. Rev. Comput. Mol. Sci.* **7**, e1298 (2017).

17. Rhoads, D. G. & Lowenstein, J. M. Initial velocity and equilibrium kinetics of myokinase. *J. Biol. Chem.* **243**, 3963–3972 (1968).

18. Rogne, P. *et al.* Nucleation of an activating conformational change by a cation-π interaction. *Biochemistry* **58**, 3408–3412 (2019).

19. Delaglio, F. *et al.* NMRPipe: A multidimensional spectral processing system based on UNIX pipes. *J. Biomol. NMR 1995 63* **6**, 277–293 (1995).

20. Helgstrand, M., Hard, T. & Allard, P. Simulations of NMR pulse sequences during equilibrium and non-equilibrium chemical exchange. *J. Biomol. NMR 2000 181* **18**, 49–63 (2000).

21. Lee, W., Tonelli, M. & Markley, J. L. NMRFAM-SPARKY: Enhanced software for biomolecular NMR spectroscopy. *Bioinformatics* **31**, 1325–1327 (2015).

22. Goddard, T. D. & Kneller, D. G. SPARKY 3. (University of California, San Francisco).

23. Wolf-Watz, M. *et al.* Linkage between dynamics and catalysis in a thermophilic-mesophilic enzyme pair. *Nat. Struct. Mol. Biol.* **11**, 945–949 (2004).

24. Rogne, P. *et al.* Structural basis for GTP versus ATP selectivity in the NMP kinase AK3. *Biochemistry* **59**, 3570–3581 (2020).

25. Selvaratnam, R. *et al.* The projection analysis of NMR chemical shifts reveals extended EPAC autoinhibition determinants. *Biophys. J.* **102**, 630–639 (2012).

26. Müller, C. W., Schlauderer, G. J., Reinstein, J. & Schulz, G. E. Adenylate kinase motions during catalysis: an energetic counterweight balancing substrate binding. *Structure* **4**, 147–156 (1996).

27. Tait, C. E. & Stoll, S. Coherent pump pulses in Double Electron Electron Resonance spectroscopy. *Phys. Chem. Chem. Phys.* **18**, 18470–18485 (2016).

28. Stoll, S. & Schweiger, A. EasySpin, a comprehensive software package for spectral simulation and analysis in EPR. *J. Magn. Reson.* **178**, 42–55 (2006).

29. Worswick, S. G., Spencer, J. A., Jeschke, G. & Kuprov, I. Deep neural network processing of DEER data. *Sci. Adv.* **4**, eaat5218 (2018).

30. Keeley, J. *et al.* Neural networks in pulsed dipolar spectroscopy: a practical guide.

31. Jeschke, G., Koch, A., Jonas, U. & Godt, A. Direct Conversion of EPR Dipolar Time Evolution Data to Distance Distributions. *J. Magn. Reson.* **155**, 72–82 (2002).

32. Jeschke, G. Distance measurements in the nanometer range by pulse EPR. *Chem. Phys. Chem.* **3**, 927–932 (2002).

33. Jeschke, G. *et al.* DeerAnalysis2006 - a comprehensive software package for analyzing pulsed ELDOR data. *Appl. Magn. Reson* **30**, 473–498 (2006).

34. Fábregas Ibáñez, L., Jeschke, G. & Stoll, S. DeerLab: a comprehensive software package for analyzing dipolar electron paramagnetic resonance spectroscopy data. *Magn. Reson* **1**, 209–224 (2020).

35. Edwards, T. H. & Stoll, S. Optimal Tikhonov Regularization for DEER Spectroscopy. *J. Magn. Reson.* (2018). doi:10.1016/j.jmr.2018.01.021

36. Reinstein, J. *et al.* Fluorescence and NMR investigations on the ligand binding properties of adenylate kinases. *Biochemistry* **29**, 7440–7450 (1990).

37. Hansia, P., Guruprasad, N. & Vishveshwara, S. Ab initio studies on the tri- and diphosphate fragments of adenosine triphosphate. *Biophys. Chem.* **119**, 127–136 (2006).

38. Simanshu, D. K., Savithri, H. S. & Murthy, M. R. N. Crystal structures of Salmonella typhimurium propionate kinase and its complex with Ap4A: Evidence for a novel Ap4A synthetic activity. *Proteins* 1379–1388 (2008). doi:10.1002/prot

39. Müller, C. W. & Schulz, G. E. Structure of the complex between adenylate kinase from Escherichia coli and the inhibitor Ap5A refined at 1.9 Å resolution. A model for a catalytic transition state. *J. Mol. Biol.* **224**, 159–177 (1992).

40. Ojeda-May, P. *et al.* Dynamic connection between enzymatic catalysis and collective protein motions. *Biochemistry* **60**, 2246–2258 (2021).

41. Matthews, B. W. Solvent content of protein crystals. *J. Mol. Biol.* **33**, 491–497 (1968).

42. Kantardjieff, K. A., Rupp, B. & Keck, W. M. Matthews coefficient probabilities: Improved estimates for unit cell contents of proteins, DNA, and protein-nucleic acid complex crystals. *Protein Sci.* **12**, 1865–1871 (2003).

43. Ådén, J., Verma, A., Schug, A. & Wolf-Watz, M. Modulation of a pre-existing conformational equilibrium tunes adenylate kinase activity. *J. Am. Chem. Soc.* **134**, 16562–16570 (2012).

44. Hagelueken, G., Ward, R., Naismith, J. H. & Schiemann, O. MtsslWizard: In Silico Spin-Labeling and Generation of Distance Distributions in PyMOL. *Appl. Magn. Reson.* **42**, 377–391 (2012).

45. Gong, Z., Gu, X.-H., Guo, D.-C., Wang, J. & Tang, C. Protein Structural Ensembles Visualized by Solvent Paramagnetic Relaxation Enhancement. *Angew. Chemie Int. Ed.* **56**, 1002–1006 (2017).

46. Ådén, J. & Wolf-Watz, M. NMR identification of transient complexes critical to adenylate kinase catalysis. *J. Am. Chem. Soc.* **129**, 14003–14012 (2007).
